# Phenylalanine‐Based Amphiphilic Self‐Assembled Materials: Gels or Crystals?

**DOI:** 10.1002/chem.202404586

**Published:** 2025-03-17

**Authors:** Fabia Cenciarelli, Demetra Giuri, Silvia Pieraccini, Stefano Masiero, Simone D'Agostino, Claudia Tomasini

**Affiliations:** ^1^ Dipartimento di Chimica Giacomo Ciamician Università di Bologna Via Piero Gobetti, 85 40129 Bologna Italy

**Keywords:** crystals, hydrogels, low molecular weight gelators, phenylalanine-based amphiphiles, supramolecular materials

## Abstract

We prepared three simple molecules, that we chose as representative examples of amphiphilic and bolamphiphilic amino acid derivatives: *N*‐lauroyl‐L‐phenylalanine (Lau‐Phe‐OH), *N*‐palmitoyl‐L‐phenylalanine (Pal‐Phe‐OH), *N,N*‐azeloyl‐L‐diphenylalanine Az‐(Phe‐OH)_2_, to study the influence of the aliphatic side chain on the formation of supramolecular materials. We found that Pal‐Phe‐OH is a very efficient gelator in contrast with Az‐(Phe‐OH)_2_ that efficiently forms crystals, while Lau‐Phe‐OH forms metastable hydrogels that slowly become crystals. We demonstrated by X‐ray diffraction that Lau‐Phe‐OH and Pal‐Phe‐OH easily form hetero‐intermolecular hydrogen bonds between the carboxylic and amidic groups, while Az‐(Phe‐OH)_2_ forms homo‐intermolecular hydrogen bonds, i. e., the typical carboxylic ring dimer and chains between the amidic functions, which leads to an extended and robust 2D hydrogen bonding network. Moreover, Lau‐Phe‐OH is more ordered than Pal‐Phe‐OH and the comparison of these results clearly indicates that the reduced order of Pal‐L‐Phe‐OH is the main reason for the efficiency of this molecule as supergelator.

## Graphical abstract

Pal‐Phe‐OH is a very efficient gelator in contrast with Az‐(Phe‐OH)_2_ that efficiently forms crystals, while Lau‐Phe‐OH forms metastable hydrogels that slowly become crystals. X‐ray diffraction analysis demonstrate that Lau‐Phe‐OH and Pal‐Phe‐OH easily form hetero‐intermolecular hydrogen bonds between the carboxylic and amidic groups, while Az‐(Phe‐OH)_2_ forms homo‐intermolecular hydrogen bonds, which leads to an extended and robust 2D hydrogen bonding network.

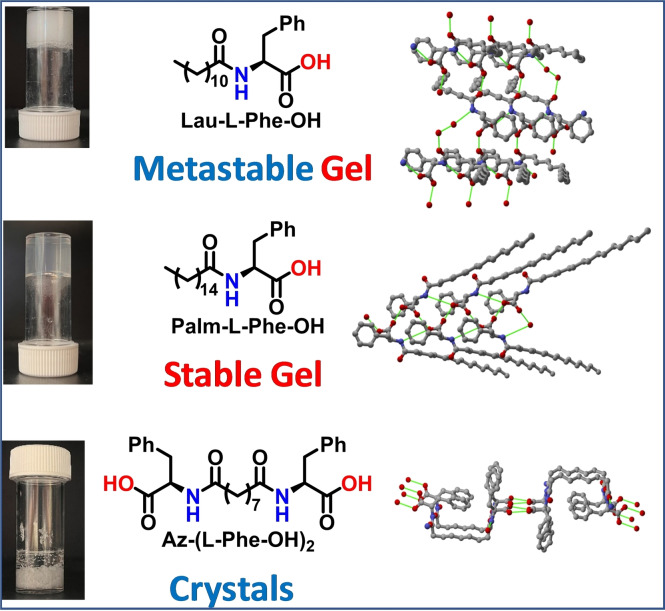

## Introduction

Low‐molecular‐weight gelators (LMWGs) are small molecules capable of forming supramolecular materials[^1^, ^2^] through the assembly driven by multiple non‐covalent interactions, including π‐π stacking, hydrogen and halogen bonding,[^3^, ^4^, ^5^] van der Waals forces, and electrostatic interactions.[^6^, ^7^] These interactions typically rely on the partial solubility of the gelator molecules in a selected solvent system.[^8^, ^9^] In recent years, peptide derivatives have garnered significant attention as LMWGs due to their inherent chemical and functional versatility. This versatility allows for easy structural modification by altering the number and sequence of amino acids and the inclusion of different protecting groups, which in turn enables fine‐tuning of the material properties.^[10]^ Additionally, peptide‐based gelators benefit from a wide range of non‐covalent interactions, are often biocompatible *in vivo*, and offer adjustable proteolytic stability depending on the chemical structure and configuration of the amino acids.[^11^, ^12^, ^13^] Due to these characteristics, peptide‐based LMWGs have been extensively explored as delivery carriers,[^13^, ^14^, ^15^, ^16^, ^17^] as they offer greater molecular‐scale manipulation compared to synthetic polymers, while also providing tunable viscoelastic and mechanical properties. Gels formed by these gelators are soft materials, primarily composed of a solvent (water in the case of hydrogels or organic solvents in organogels) or a solvent mixture, supported by a three‐dimensional network of entangled fibers.

Phenylalanine (Phe) and its derivatives have gained increasing attention in the field of LMWGs due to their ability to form stable supramolecular structures.^[9]^ The aromatic side chain of Phe facilitates molecular self‐assembly, leading to the formation of gels with specific physicochemical properties. Numerous studies have demonstrated the effectiveness of Phe derivatives as gelators, highlighting their potential in biomedical and technological applications.[^18^, ^19^] For instance, *N*‐protected Phe derivatives, such as those protected with the Fmoc group, have been extensively studied for their ability to form biocompatible hydrogels with potential applications in drug delivery and tissue engineering.[^20^, ^21^, ^22^] Ulijn et al.^[23]^ demonstrated that Fmoc‐Phe derivatives can self‐assemble into nanofibers that form hydrogels capable of encapsulating and releasing therapeutic agents in a controlled manner.[^24^, ^25^] Another study by Smith et al.^[26]^ explored the self‐assembly of Phe‐based peptides into nanofibers, generating hydrogels with tunable mechanical properties, suggesting their potential utility in tissue engineering applications.

With the aim of replacing Fmoc, which may be somehow toxic,[^27^, ^28^] in this project we strategically designed a family of three gelators incorporating fatty acids and the aromatic amino acid residue. The design of amphiphiles structures was a deliberate choice due to their unique molecular structure, featuring a hydrophilic head and a hydrophobic tail. Amphiphiles are well‐known for their self‐aggregating properties^[29]^ and their ability to impart thermo‐responsiveness and enhanced mechanical strength to the systems in which they are used.[^30^, ^31^]

Lauric acid (C12), palmitic acid (C16), and azelaic acid (C9) are exemplary molecules within this category. Azelaic acid, a natural dicarboxylic acid found in wheat, rye, and barley, is industrially produced via the ozonolysis of oleic acid and serves as a precursor for various industrial products, including polymers and plasticizers. In our research group, azelaic acid has been widely used for its ability to form bolaamphiphilic structures,[^32^, ^33^, ^34^, ^35^] which are promising candidates for gel formation.[^36^, ^37^, ^38^]

Using liquid phase synthesis, we prepared three simple molecules, each containing L‐Phe and a fatty acid, that we chose as representative examples of amphiphilic and bolamphiphilic amino acid derivatives: *N*‐lauroyl‐L‐phenylalanine (Lau‐Phe‐OH) **A**, *N*‐palmitoyl‐L‐phenylalanine (Pal‐Phe‐OH) **B**, *N,N*‐azeloyl‐L‐diphenylalanine Az‐(Phe‐OH)_2_ **C**, to study the influence of the aliphatic side chain on the formation of supramolecular materials.

We incorporated these amphiphilic units into our design, anticipating that the methylene groups in their backbone would confer increased hydrophobicity to the material. Some of these compounds have already been reported in the past and their behaviour has been partially described.[^39^, ^40^]

This work pursues two primary objectives. First, it aims to identify a biocompatible gelator and comprehensively investigate its hydrogel‐forming capabilities, particularly at low concentrations. Second, and more importantly, this study seeks to rationalize the gelator‘s behavior based on its chemical structure. Subtle variations in molecular structure can significantly impact self‐assembly, ultimately altering the physical properties of the resulting supramolecular material.

## Results and Discussion

**Figure 1:**
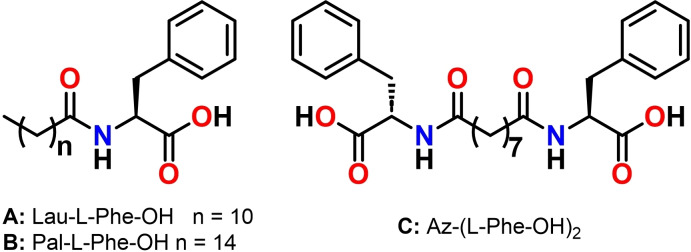
The chemical structures of the molecules described in this work.

Compounds **A**, **B** and **C** have been prepared in multigram scale, starting from commercially available L‐Phe‐OMe, lauric acid, palmitic acid and azelaic acid, through two simple steps in overall yields ranging from 71 % to 78 % (Figure 1, Scheme S1 and S2 and Experimental). All the molecules are white solids.

As we want to form hydrogels using the pH variation method, it is crucial to know at what pH the protonation of the carboxylates takes place, as carboxylates are usually soluble in water, while the corresponding carboxylic acids are not. Indeed, the aggregation in basic solution of amphiphilic and bolamphiphilic carboxylates may lead to apparent pK_*a*_s depending on concentration,[^41^, ^42^, ^43^, ^44^] which may be very different from the pK_*a*_ of fully soluble carboxylic acid (*i. e*., acetic acid). This is why we first performed the measurement of the pK_*a*_.

**Figure 2:**
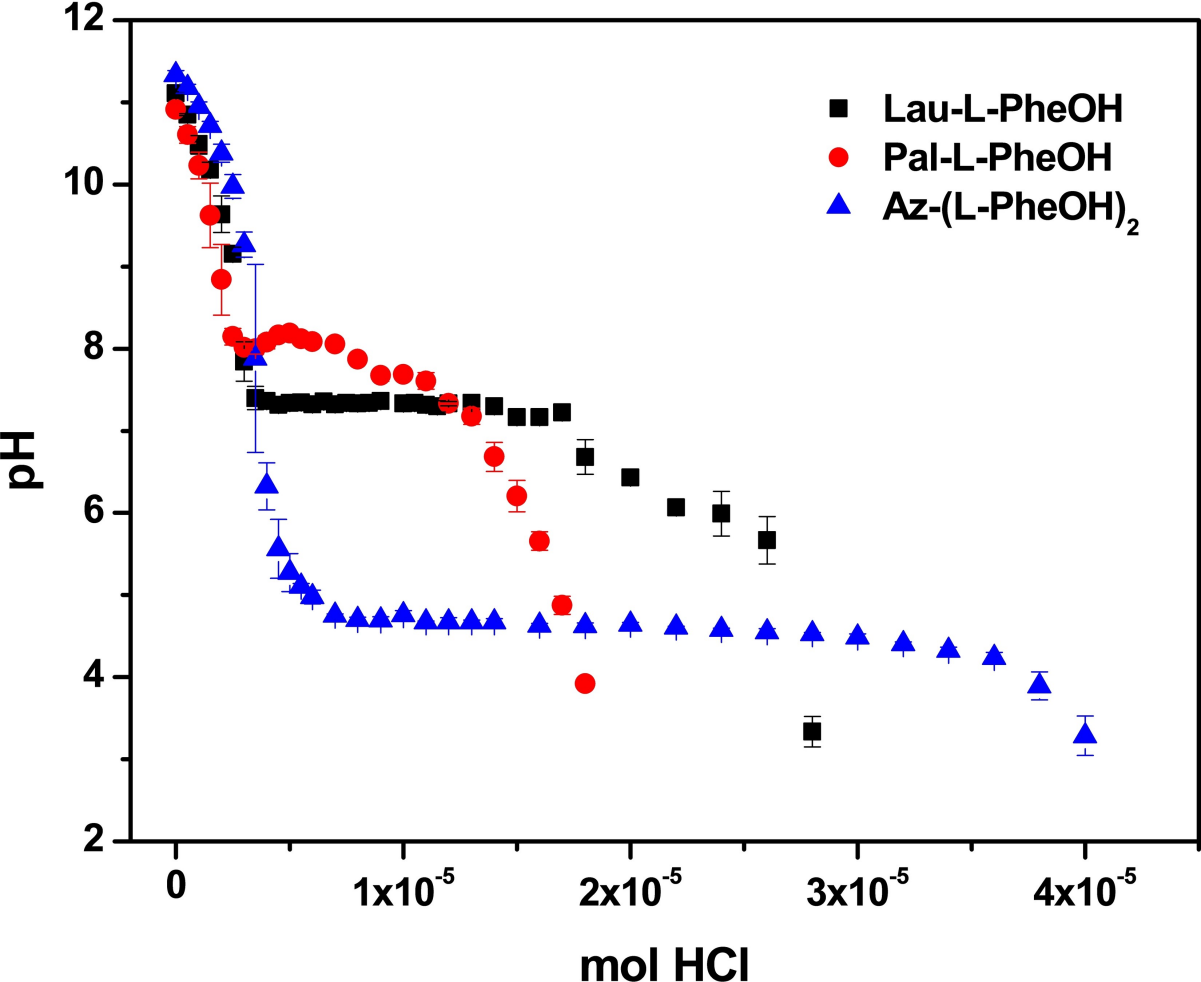
Titration curves (pH versus mol of added HCl 0.1 M) of the following compounds: Lau‐Phe‐OH **A** (0.5 % w/V, 14.38 mM), Pal‐Phe‐OH **B** (0.5 % w/V, 12.38 mM), Az‐(Phe‐OH)_2_ **C** (0.5 % w/V, 10.36 mM). The experiments were repeated in triplicate and results are expressed as mean ± standard deviation.

Figure 2 summarizes the results that we obtained with titration of the three molecules in a 0.5 % w/v concentration from pH 11 to pH 3, adding aliquots of a 0.1 M HCl solution. All the experiments were run in triplicate. The pK_a_ was defined as the pH at which 50 % of the gelator molecules are protonated. In most cases, this pK_a_ value corresponded to a plateau in the pH titration curve (Table S1).

While amphiphilic Lau‐Phe‐OH **A** and Pal‐Phe‐OH **B** show an apparent pK_*a*_ above the expected position/value, respectively at pH 7.3 and 7.7, bolamphipilic **C** shows pK_*a*_ at 4.6, very similar to fully soluble acetic acid. This outcome suggests that Lau‐Phe‐OH **A** and Pal‐Phe‐OH **B** form micelles, due to the presence of the aliphatic portion. This is not true for Az‐(Phe‐OH)_2_ **C**, probably due to the presence of two carboxylic groups, that balance the aliphatic chain. The calculation of log*P* values, expressing lipophilicity, is in agreement with this trend, as reported in Table 1. Log*P* is the log of the partition coefficient of a solute between octanol and water, at near infinite dilution and is widely used in drug discovery and development as an indicator of potential utility of a solute as a drug.^[45]^ Log*P* of Az‐(Phe‐OH)_2_ **C** is very low, thus confirming its high hydrophilicity, while the log*P* of Lau‐Phe‐OH **A** and Pal‐Phe‐OH **B** is considerably higher, accounting for their hydrophobicity.

**Table 1:** Comparison between logP and apparent pKa of gelators Lau‐Phe‐OH **A**, Pal‐Phe‐OH **B**, and [Az‐(Phe‐OH)_2_] **C**.

Gelator	log*P*	pK_a_
A	4.48	7.3
B	6.50	7.7
C	0.75	4.6

To better understand the effect of the chemical structure on the gelation attitude of the three molecules, a further analysis of the packing of the molecules was obtained by crystallization of Lau‐Phe‐OH **A**, Pal‐Phe‐OH **B**, and Az‐(Phe‐OH)_2_ **C**.

**Figure 3:**
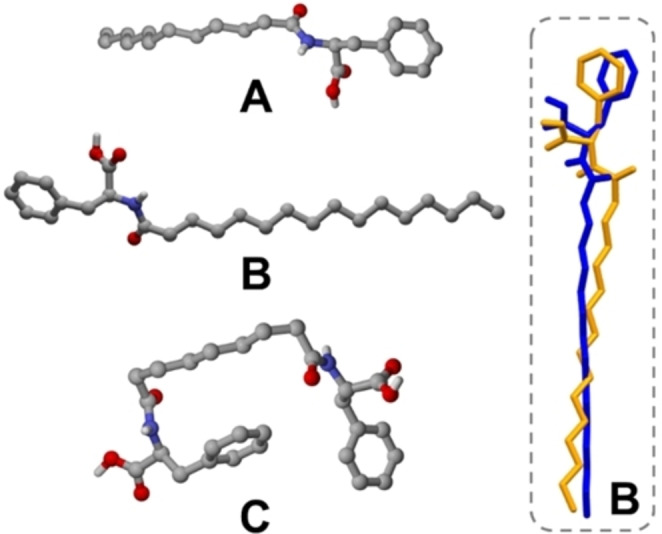
Molecular structures of compounds Lau‐Phe‐OH **A**, Pal‐Phe‐OH **B** and Az‐(Phe‐OH)_2_ **C**, as determined via XRD analysis. Inset: overlay diagram showing the different conformation of the two independent molecules found within crystalline **B**. H_CH_ atoms omitted for clarity.

Several attempts of crystallization have been performed in methanol, ethanol, isopropanol, acetonitrile, acetonitrile/toluene and isopropanol/toluene mixtures. Crystals suitable for X‐ray diffraction measurements were grown from methanol and ethanol for compounds Lau‐Phe‐OH **A** and Az‐(Phe‐OH)_2_ **C**, respectively, while compound Pal‐Phe‐OH **B**, despite many efforts, was only obtained invariably in polycrystalline form. Consequently, its structure determination was attempted using powder XRD data, with the help of TGA analysis, which ruled out the presence of solvates/hydrates (see Experimental Section for details). Structural XRD analysis confirmed the molecular structure for each synthesized compound (Figure 3) and as shown below revealed details concerning their intermolecular interaction patterns.

**Figure 4:**
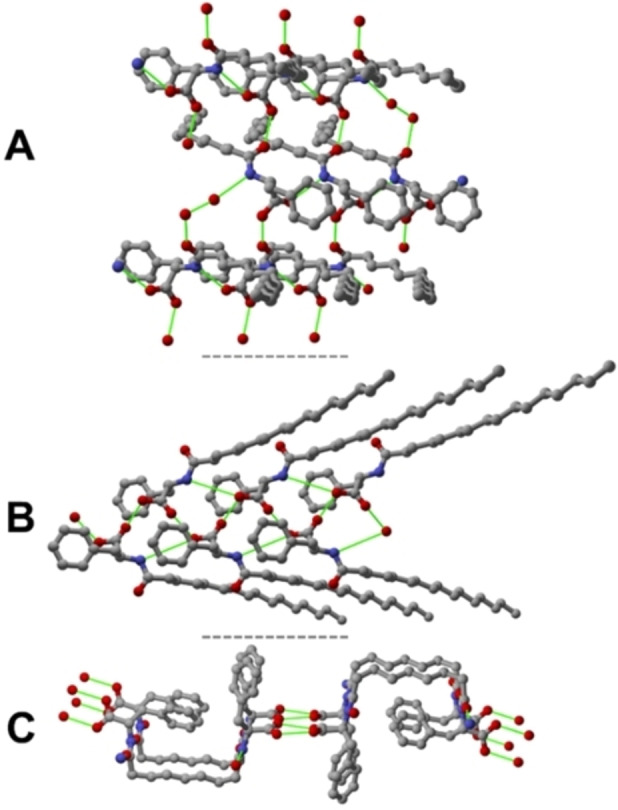
The intermolecular hydrogen bonding interactions detected within crystalline Lau‐Phe‐OH **A**, Pal‐Phe‐OH **B** and Az‐(Phe‐OH)_2_ **C**. H‐atoms omitted for clarity; hydrogen bonding interactions are depicted in green.

Lau‐Phe‐OH **A** crystallizes in the orthorhombic system and with the P2_1_2_1_2_1_ space group (details in Table S2), with one molecule in the asymmetric unit. Within the crystal, molecules of Lau‐Phe‐OH **A** engage in multiple intermolecular hydrogen bonding interactions between N‐ and O‐ atoms from amide and carboxylic functions, respectively, to form a chain [N_N—H_⋅⋅⋅O_C=O_ = 3.035(7) Å]. Additionally, orthogonal hydrogen bonding interactions involving the O‐atoms from carboxylic and amide functions [O_COOH_⋅⋅⋅O_C=O_ = 2.570(6) Å] are present. Overall, a 3D hydrogen‐bonded net is obtained (Figure 4).

Conversely, Pal‐Phe‐OH **B** crystallizes in the monoclinic system with the P2_1_ space group (details in Table S1). Based on the unit cell volume and a multiplicity of Z=2, the asymmetric unit is expected to contain three independent molecules (Z’=3), as the molecular and cell volume were estimated to be around 600 Å, and 3500 Å, respectively. Despite extensive efforts, the structural solution yielded a reliable model that excludes the third molecule from the asymmetric unit. We can only hypothesize that this discrepancy arises from significant crystallographic disorder affecting this molecule, which could not be adequately addressed using powder data.

Within the crystal, each independent molecule of Pal‐Phe‐OH **B** shows a slightly different conformation (Figure 3‐inset) and establishes intermolecular hydrogen bonds with symmetry‐generated molecules leading to endless chains, as shown in Figure 4. The framework can be described as an elongated arrangement where the lipophilic chains point outwards from the aromatic core. These hydrogen bonding interactions involve the N‐ and the O‐atoms from the amide and carboxylic functions [N_N—H_⋅⋅⋅O_C=O_= 3.117(2) Å], and the O‐atoms from carboxylic functions [O_COOH_ ⋅⋅⋅O_C=O_ = 2.619(9)‐ 2.713(9) Å].

Az‐(Phe‐OH)_2_ **C** crystallizes in the monoclinic system and with the C_2_ space group (details in Table S2), with one molecule in the asymmetric unit. Both carboxylic functions exhibited crystallographic disorder that was treated over two positions (see experimental section for details) and engage hydrogen bonding leading to the typical ring dimer [O_COOH_⋅⋅⋅O_COOH_= 2.576(7), 2.691(1) Å], at the same time the amidic functions form intermolecular hydrogen bonding interactions between the N‐ and O‐atoms [N_N—H_⋅⋅⋅O_C=O_ = 2.933(6), 2.964(6) Å]. As a result, a 2D hydrogen‐bonded network is obtained (Figure 4).

While Lau‐Phe‐OH **A** and Pal‐Phe‐OH **B** easily form hereto‐intermolecular interactions between the amidic and carboxylic functions leading to 3D and 1D networks, as demonstrated by the analysis of the crystal packing, Az‐(Phe‐OH)_2_ **C** forms an extended 2D hydrogen bonding network held together by homo‐intermolecular interactions, namely the typical ring dimer formed between the carboxylic groups and the hydrogen bonds between the amidic functions. These different types of interactions, *i. e*., hetero‐ vs homo‐ are expected to act differently in terms of crystal stabilization. To evaluate the magnitude of such contributions, intermolecular interaction energy (IIEs)[^46^, ^47^, ^48^] analyses were performed using the CrystalExplorer software package.[^47^, ^48^] It is worth pointing out that such a calculation could not be performed for **B** because only a partial structure was obtained for this compound.

For crystalline **A** IIEs calculations indicate that the first hydrogen bonded neighbours interact with a total energy of −59.6 and −63.2 kJ⋅mol^−1^. As expected, given the electrostatic nature of the hydrogen bonds between the COOH and CONH groups, the IIEs are dominated by the electrostatic component of the total energy (Figure S4 and Table S3). On the other hand, for crystalline **C** stronger IIEs were found, *i. e*., −140.1 kJ⋅mol^−1^ and −118.5 kJ⋅mol^−1^, for neighbouring molecules forming the carboxylic dimer and hydrogen bonds between amide functions, respectively. Again, the electrostatic nature of the homo‐hydrogen bonds dominates over the total energy (Figure S4 and Table S2). Therefore, compound **C** seems more likely to form stable crystalline phases rather than gel materials, which can be seen as metastable phases where crystallization is hindered or acting partially.

Holding this information, the gelation activity of the three molecules has been tested with a standard procedure. The first attempts were done with a 0.5 % v/w gelator concentration. The gelator was suspended in Milli‐Q® H_2_O, a small excess of a 1 M aqueous solution of NaOH was added, then the solution was stirred and sonicated until complete dissolution of the gelator. To trigger the formation of the gel, we added glucono‐δ‐lactone (GdL) to reach a final pH ~4 and immediately gently swirled the mixture to allow the dissolution of the trigger.^[49]^ This pH is well below the pK_*a*_ of the three molecules, so ensuring that we reached a pH where the molecules are fully protonated.

As foreseen from the pK_*a*_ and X‐ray diffraction analysis, gelators Lau‐Phe‐OH **A** and Pal‐Phe‐OH **B**, that aggregate in solution, form strong gels, while Az‐(Phe‐OH)_2_ **C** does not. The results obtained with X‐ray diffraction analysis accounts for this outcome, as Az‐(Phe‐OH)_2_ **C** forms hydrogen bonding, but no hydrophobic interactions that usually favour the formation of gels. Thus, the study on the hydrogel properties was concentrated on Lau‐Phe‐OH **A** and Pal‐Phe‐OH **B**.

To check and compare the mechanical properties of the supramolecular hydrogels from Lau‐Phe‐OH **A** and Pal‐Phe‐OH **B**, we prepared some hydrogels using both gelators in 0.5 % v/w concentration.[^50^, ^51^, ^52^] The rheological measurements were performed using an Anton Paar (Graz, Austria) MCR102 rheometer. The gels were directly prepared in the Sterilin Cups®, which fit in the rheometer. Oscillatory amplitude sweep experiments (γ: 0.01–100 %) were performed in triplicate at 23 °C using a constant angular frequency of 10 rad/s, 16 h after the addition of trigger, to allow a complete gel formation.

**Figure 5:**
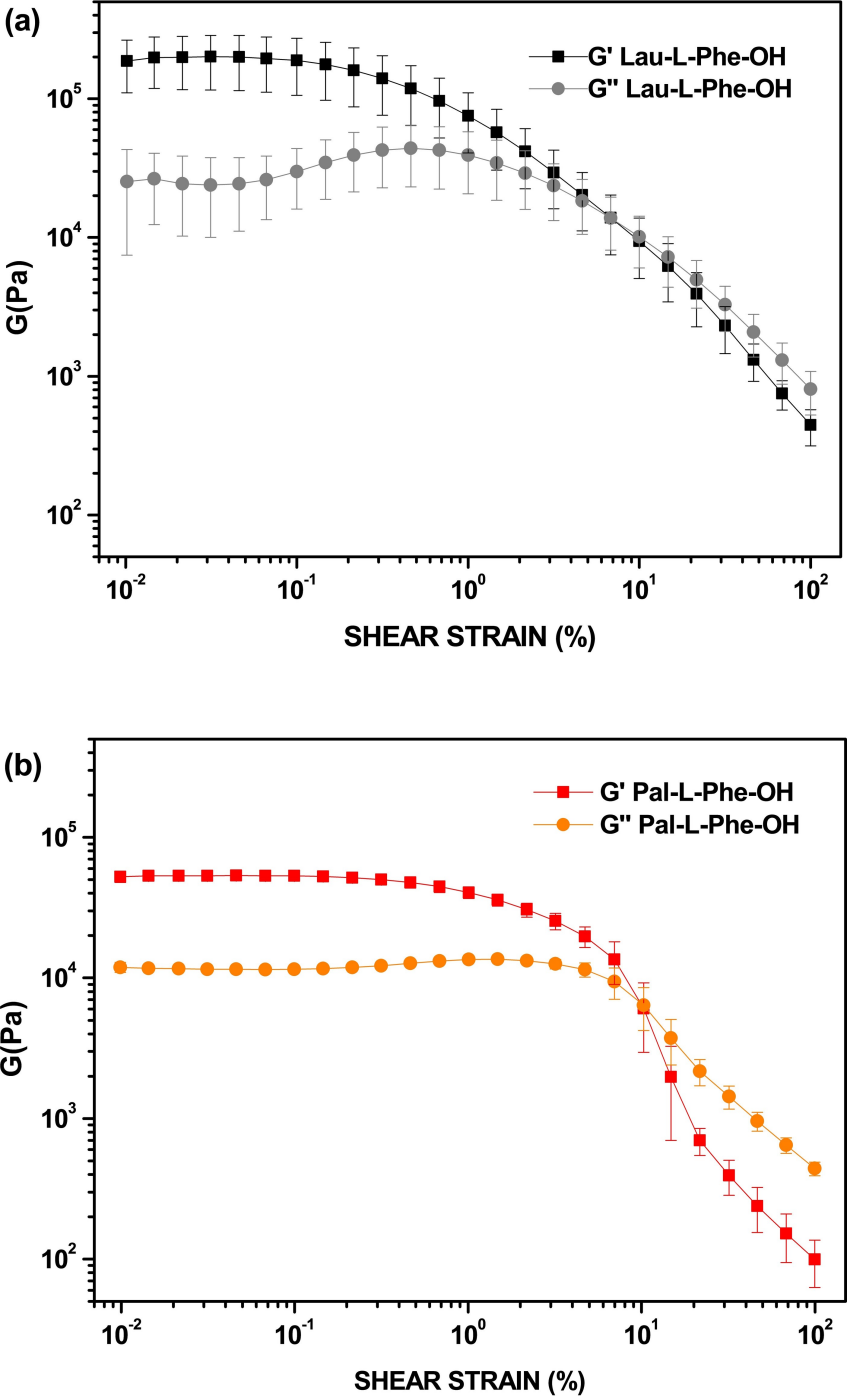
Amplitude sweep test of the hydrogels at 0.5 % w/V. (a): Lau‐L‐Phe‐OH **A**; (b): Pal‐L‐Phe‐OH **B**. The experiments were repeated in triplicate and results are expressed as mean ± standard deviation. Oscillatory amplitude sweep experiments (γ: 0.01−100 %) were performed at 23 °C using a constant angular frequency of 10 rad/s.

**Figure 6:**
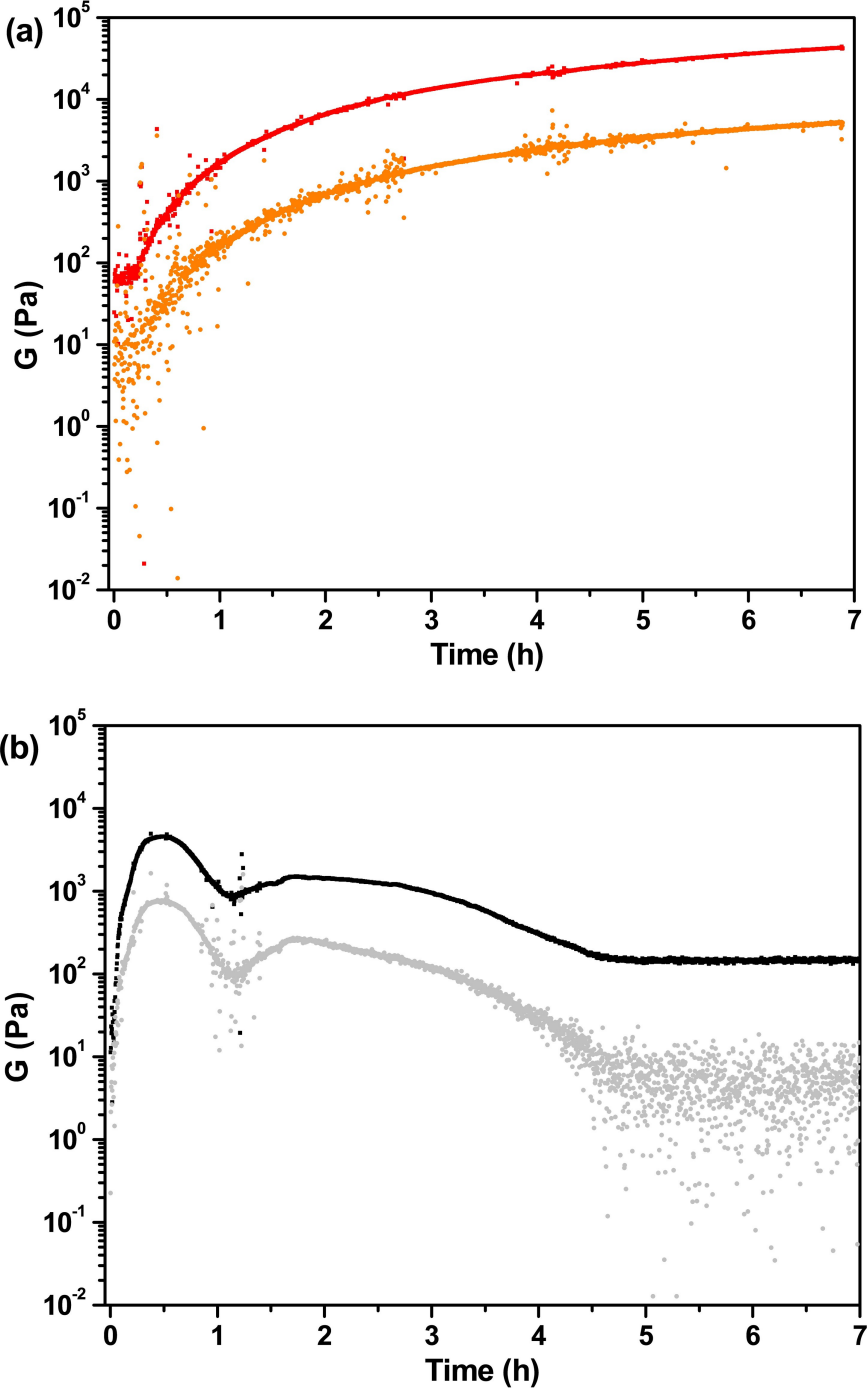
Time sweep test of the hydrogels at 0.5 % w/V. (a): Pal‐L‐Phe‐OH **B** (G’=red, G”=orange); (b): Lau‐L‐Phe‐OH **A** (G’=black, G”=grey). Oscillatory amplitude sweep experiments (γ: 0.01−100 %) were performed at 23 °C using a constant angular frequency of 10 rad/s.

Both gels are very strong as they have a storage modulus ranging between 10^4^ and 10^5^ Pascal, although the data of Lau‐Phe‐OH **A** are affected by a high error, thus suggesting that they are slightly reproducible (Figure 5). To assess the gels formation and behaviour over time, we recorded a time sweep for 7 hours (Figure 6). This outcome clearly shows that the gelification processes of the two hydrogels is very different: hydrogel from Pal‐Phe‐OH **B** is totally stable and increases its strength even in the presence of the shaft. In contrast, hydrogel from Lau‐Phe‐OH **A** is not stable with time and immediately reduces its strength. The gel may last for longer times (several days) if not perturbated by the shaft, but the final result is the same. The tendency to form crystal of Lau‐Phe‐OH **A** makes the corresponding gels less stable.

To gain insights into the relationship between the gel, aerogel, and the crystal structure, powder XRD analyses were conducted. The two aerogels were prepared by soaking freshly prepared samples of hydrogels in liquid nitrogen to promote a fast freezing of the gel, followed by freeze‐drying until the complete removal of water.

**Figure 7:**
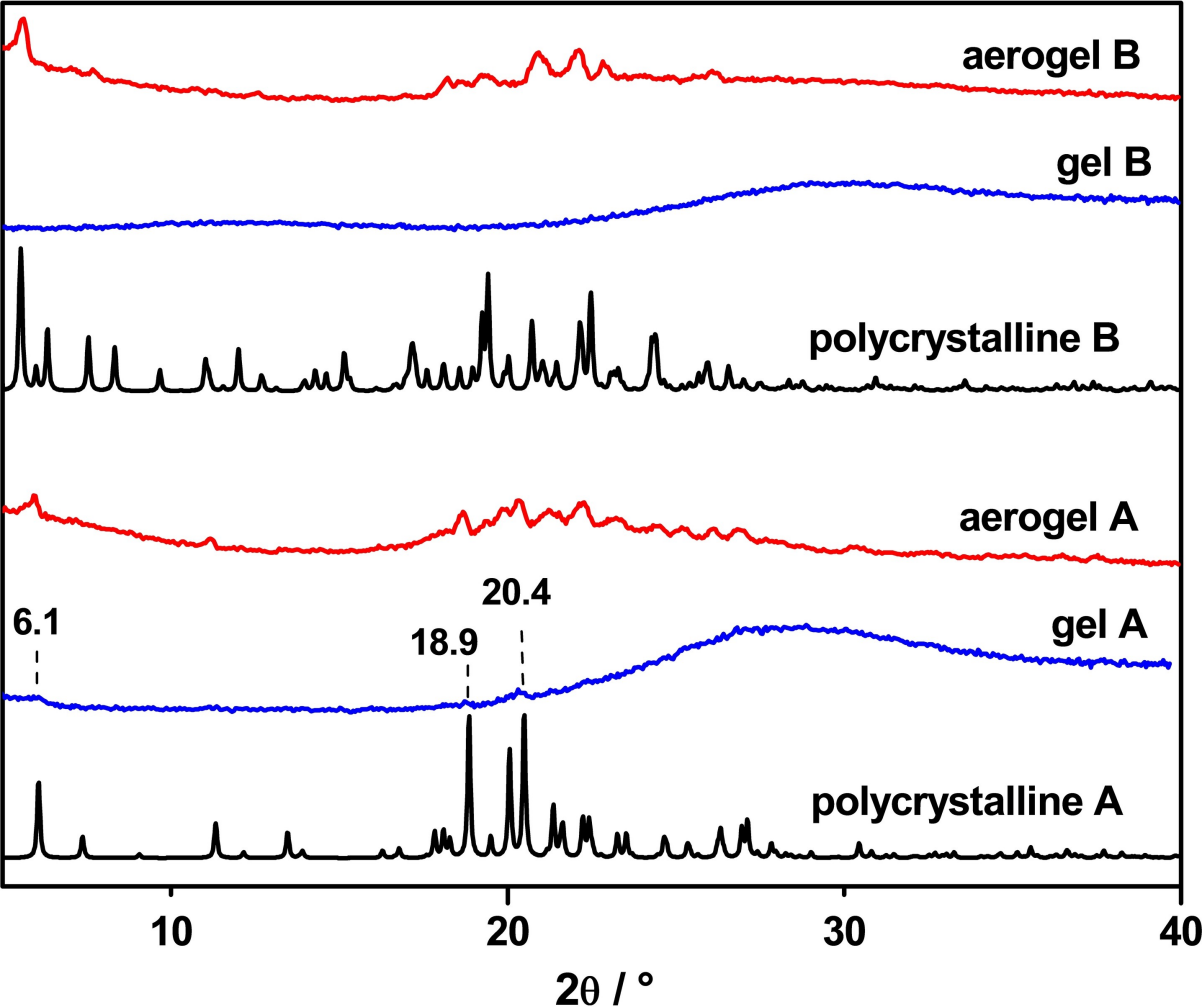
Comparison between the experimental XRD patterns recorded at RT for polycrystalline (black‐line), gel (blue‐line), and aerogel (red‐line) samples of compounds **A** and **B**.

In the diffractogram of the gel obtained from compound **A**, along with the amorphous halo, three weak and broadened diffraction peaks at low angles (6.1°, 18.9°, and 20.4°) are detectable, indicating the presence of a crystalline phase in the sample, whereas, the patterns of the polycrystalline sample and aerogels match well, indicating, thus, that the aerogel and gelator **A** crystals share the same structural organization (Figure 7).

On the other hand, the gel obtained from compound **B** shows only the typical halo, while those recorded on a polycrystalline sample and aerogel do not show a perfect match. This could be due to residual solvent molecules trapped inside the material and slightly altering the structural organization.

It is worth noting that during the fast‐drying process, the assembly of the gelator is unlikely to undergo drastic changes. It is much more likely that the gelator crystallizes in the same arrangement in the gel network, which is the same as or quite similar to the single crystal structure.

According to other reported studies,[^53^, ^54^, ^55^, ^56^, ^57^, ^58^] the match of the powder XRD patterns of the aerogels and bulk crystals reliably indicates that both share a similar structural organization.

**Figure 8:**
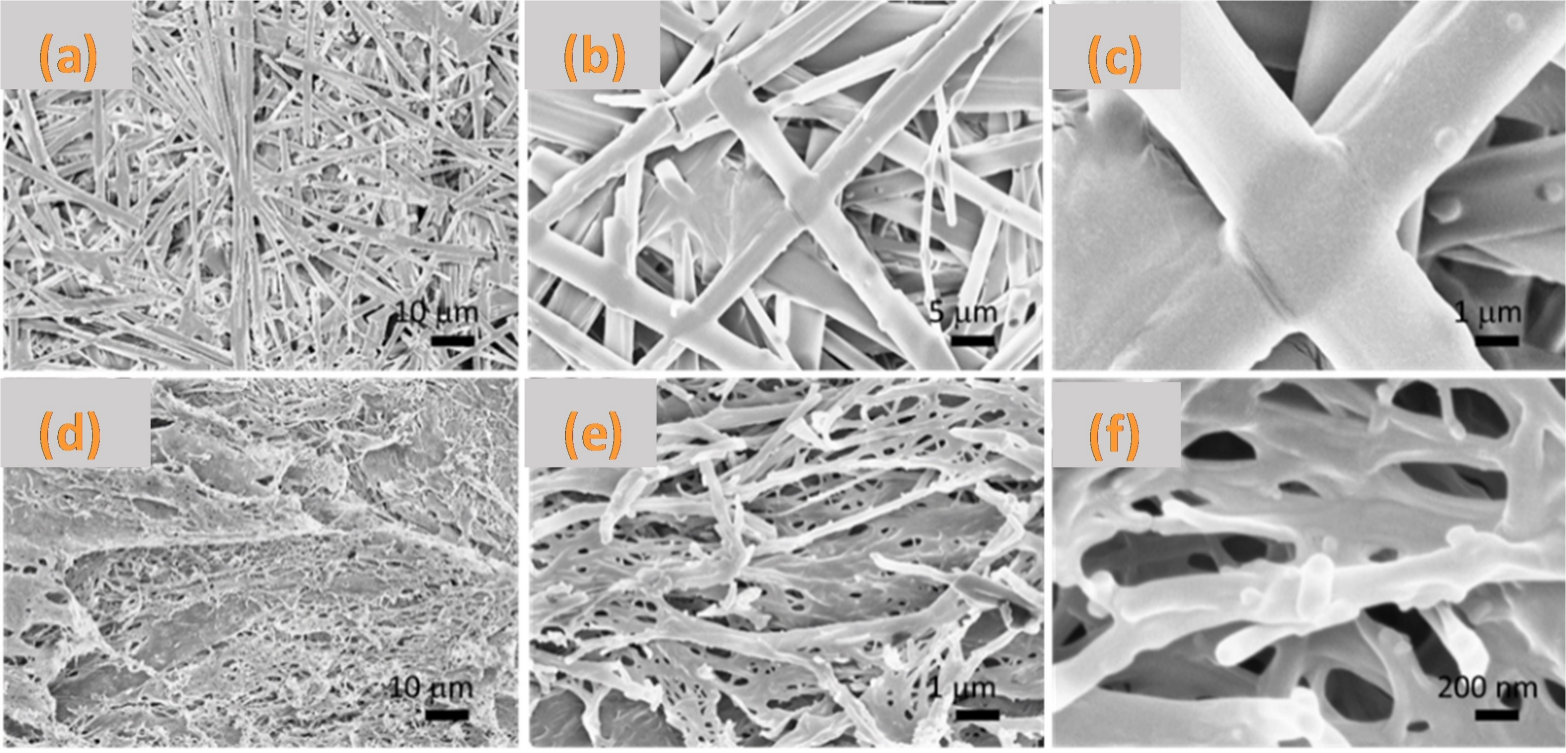
Scanning electron microscopy images of the aerogels. (a)‐(c) images show increasing magnifications of the sample Lau‐Phe‐OH **A**. (d)‐(f) images show increasing magnifications of the sample Pal‐Phe‐OH **B**.

The two aerogels were further analyzed by SEM microscopy and the obtained images are reported in Figure 8. In agreement with the above data, differences were observed between samples. Sample of Lau‐Phe‐OH **A** consists of a dense network of straight fibers with a diameter of approximately 5 μm and a length that can exceed 1 mm. Some of these fibers originate from growth centers and occasionally form bundles. The fibers frequently intersect, creating nodes, while in other areas, individual fibers are fused together to form larger diameter filaments. The material is predominantly composed of fibers, with non‐fibrous material present only in small amounts. Sample of Pal‐Phe‐OH **B** is formed of interconnected sheets with numerous holes. These sheets form a three‐dimensional structure with highly branched, fused, and bundled fibers. The diameter of these fibers is generally less than 1 μm, although it is challenging to measure it accurately due to the network complexity.

Additional information on the gelator propensity of these molecules can be obtained from the study of their minimum gelation concentration (MGC). We tested the gelation attitude of Lau‐Phe‐OH **A** and Pal‐Phe‐OH **B** reducing their concentration and the gelation attitude of Az‐(Phe‐OH)_2_ **C** increasing its concentration up to 2 % v/w (Figure S5). In any case, Az‐(Phe‐OH)_2_ **C** never formed a gel and precipitated in form of flakes.^[59]^ The minimum gelation concentration (MGC) of Lau‐Phe‐OH **A** and Pal‐Phe‐OH **B** gave very different results, as could be foreseen.

**Figure 9:**
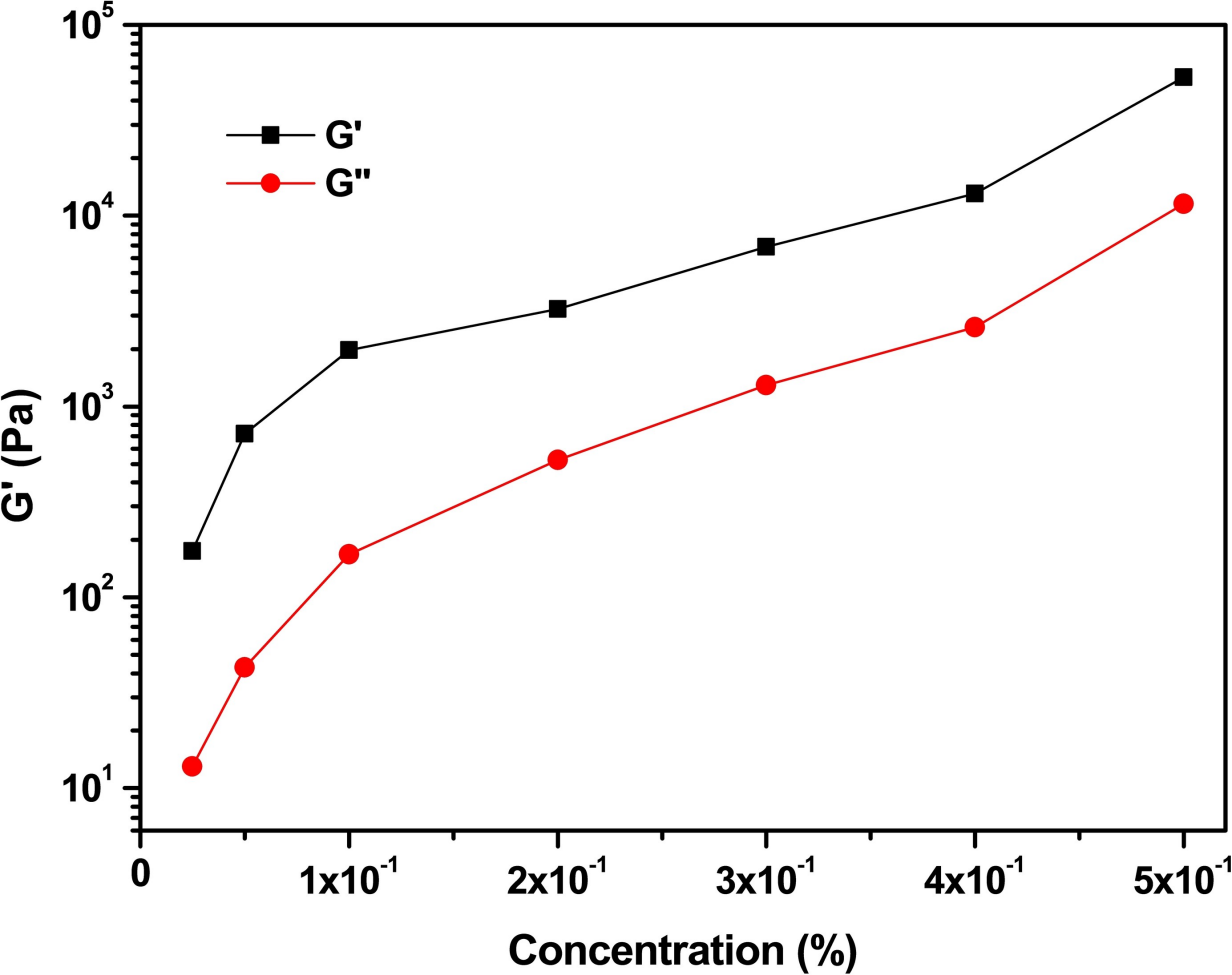
Graph obtained with the values of G’ and G” of the hydrogels of **B** as a function of the gelator concentration. The values were obtained at shear strain=0.1 % for all the samples. Oscillatory amplitude sweep experiments (γ: 0.01−100 %) were performed at 23 °C using a constant angular frequency of 10 rad/s.

Indeed, Lau‐L‐Phe‐OH **A**, that easily forms crystals, could not form any hydrogel below 0.5 % v/w concentration, while Pal‐L‐Phe‐OH **B**, that produces only small crystals in polycrystalline form, formed hydrogels at concentrations as low as 0.025 % v/w and can be defined as a super‐gelator, as previously reported for its gelation activity in PBS solution^[60]^ and in organic solvents.^[13]^ Although the strength of the **B** hydrogels is constantly reduced with the gelator concentration (Figures 9 and S6), the material is always in the gel form, as confirmed by the storage modulus G’ that is always higher than the loss modulus G”.

A further characterization of the self‐assembling gelators **A** and **B** was provided by electronic circular dichroism (ECD) spectroscopy. UV/ECD spectra were first recorded in the good solvent methanol (Figure S6). As shown by UV traces, for both compounds absorption due to the carboxyl and the amide chromophores dominates the far‐UV below 240 nm, while the aromatic band, less intense by about two orders of magnitude, is visible in the near‐UV between 300 and 240 nm.^[61]^ Corresponding ECD traces, show a monosignate positive Cotton effect at *ca*. 218 nm while the negative vibrational aromatic signals are detected in the 280–240 nm range.^[62]^ This optical activity can be congruent with inherent molecular chirality. By moving from 0.5 % w/v methanol solutions to alkaline water solutions (Figure S8), positive maxima result blue‐shifted of ca. 7 nm and signal intensities in the far‐UV region increase, likely due to micellar self‐assembly[^63^, ^64^] as suggested by the apparent pKa values. On this issue, the slightly higher molar ellipticities recorded for **A** could reflect a different degree of self‐aggregation with respect to **B**, in accordance with the longer plateau encountered in the titration curve of Figure 2. When heated to 90 °C CD intensity of both aqueous solutions decreases, consistent with possible disaggregation.

**Figure 10:**
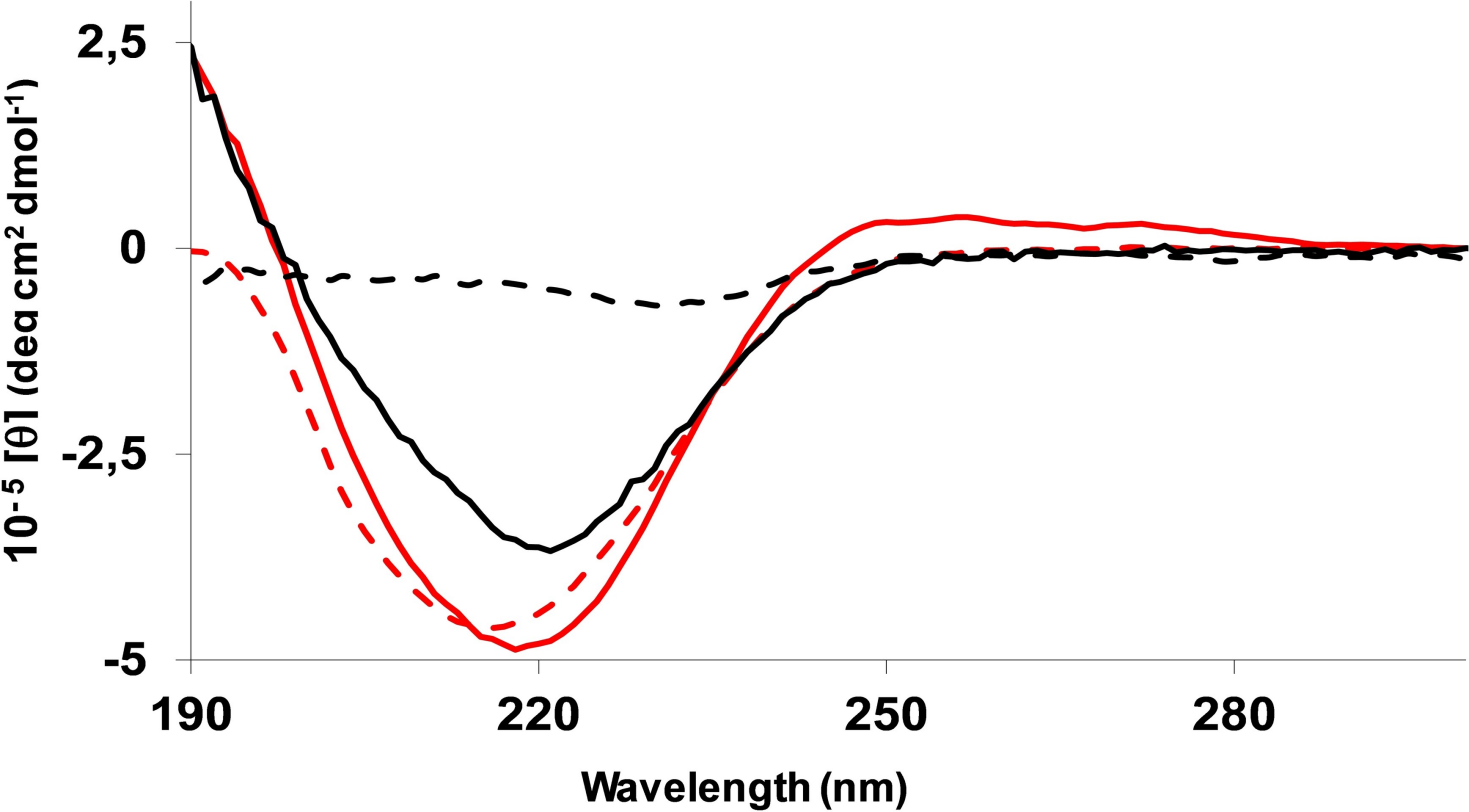
ECD spectra recorded on the 0.5 % w/v hydrogels of Lau‐Phe‐OH **A** (black) and Pal‐Phe‐OH **B** (red), 18 h (full lines) and 4 days (dashed lines) after addition of GdL to the aqueous solutions. A 0.001 cm sandwich cell was used.

As described above, the Lau‐Phe‐OH **A** and Pal‐Phe‐OH **B** basic aqueous solutions exhibit hydrogelation upon appropriate GdL addition. ECD spectra of the gel phases recorded 18 hours after the addition of GdL to 0.5 % w/v solutions are reported in Figure 10 (full lines). GdL alone does not significantly contribute to the ECD spectrum, as shown previously.^[65]^ Therefore, its contribution can be disregarded when analyzing the ECD spectra presented in Figure 10.

Under the new conditions, the ECD spectra of both **A** and **B** drastically changed (see Figure S8 for a comparison), leading to the typical ECD signature of the β‐sheet arrangement[^66^, ^67^] as seen for various peptide and short‐peptide hydrogels.[^68^, ^69^, ^70^] An intense negative band appears at about 220 nm while ellipticities become positive below 200 nm. Some differences between the two systems can be observed on intensities and band shapes. ECD of the more stable and mechanically resistant **B** hydrogel (in red) shows a minimum peak at 218 nm of significantly larger magnitude, while the weaker minimum of **A** (in black) settles at 221 nm. Moreover, after 4 days (dashed lines), the **A** hydrogel, that tends to break down with time, shows only a residual poor negative signal at 230 nm indicative of the absence of any prevalent supramolecular order. Conversely, the **B** hydrogel, supposed to reinforce over time (see above), retains *ca*. 95 % of the negative peak intensity. It is noteworthy that, in this latter case, minimum shifts to even shorter wavelengths (at 215 nm), pointing to a possible positive correlation between ECD blue‐shifting and the strength of the gel. A similar result was found by Stupp and coworkers.^61^ Indeed, at the supramolecular level, it can be considered that planar or slightly twisted β‐sheets, favouring β‐sheet packing, typically show the classical negative‐positive ECD sequence at 216–195 nm respectively,^62^ while an increase of the twisting degree, weakening attractive intermolecular interactions, generally causes red‐shifting of the exciton‐coupling.^[71]^ On these bases it can be assumed that **B** forms more planar β‐sheet like structures compared to **A**, leading to a more robust and stable hydrogel.^61^

An insight into the gelation process was given by ECD analysis performed at different times on **A** and **B** aqueous solutions after GdL addition (Figure S9). After about 1 h comparable negative ellipticities were recorded on the two samples. However, within *ca*. 2.5 hours the **A** hydrogel (in black) reached 75 % of the final ECD intensity, whereas the more stable **B** hydrogel (in red) did not reach 50 % after 4 hours. Collected data outline for the two compounds two dissimilar gelation pathways, in accordance with the time sweep test results. In particular, the more gradual self‐association observed for **B** during the first 2–3 hours is probably ascribed to supramolecular adjustments required for the more regular final organization.

## Conclusions

In this paper, we reported our effort to find a robust Phe‐based gelator that may replace Fmoc‐Phe, with the aim of eliminating the Fmoc moiety, while preserving the ability to form hydrogels. Among three molecules holding biocompatible fatty acids as side chain, we found that Pal‐Phe‐OH **B** is a very efficient gelator in contrast with Az‐(Phe‐OH)_2_ **C**, which efficiently forms crystals, and Lau‐Phe‐OH **A**, which forms metastable hydrogels that slowly become crystals.

This behaviour may be ascribed to the tendency of Lau‐Phe‐OH **A** and Pal‐Phe‐OH **B** to easily form intermolecular interactions, including extended Van der Waals interactions, while Az‐(Phe‐OH)_2_ **C** forms extended 2D network leading to the typical ring dimer, as demonstrated by the analysis of the crystal packing. Moreover, a different self‐assembly attitude for Lau‐Phe‐OH **A** and Pal‐L‐Phe‐OH **B** was demonstrated by two independent techniques: X‐ray diffraction on crystals and ECD analysis on gels.

Comparison of the obtained results indicates that the increased lipophilicity of derivative **B** confers, at different supramolecular levels, peculiar self‐correlating abilities that produce more stable systems. This property is the main reason for the efficiency of this molecule as supergelator.

This study contributes valuable insights into the prediction of molecular structures that may serve as efficient supergelators. The research suggests that the potential of a molecule as a supergelator is enhanced when it possesses specific structural features.

Firstly, the presence of stereocenters within the molecule appears to be a key factor. Stereocenters introduce chirality, which can influence the molecule ability to interact with other molecules and self‐assemble into a gel‐like structure.

Secondly, the molecule should contain functional groups capable of engaging in intermolecular interactions. These interactions, such as hydrogen bonding, van der Waals forces, or hydrophobic interactions, play a crucial role in the formation and stability of the gel network.

Furthermore, the presence of mobile moieties within the molecule is considered crucial. These mobile groups introduce a degree of disorder during the self‐assembly process. This disorder is essential to prevent the molecule from forming thermodynamically stable crystals, which would hinder gel formation. Instead, the presence of mobile moieties promotes the formation of kinetically favored gels, which are often more desirable for practical applications.

In summary, the ideal supergelator molecule should possess a combination of stereocenters, groups capable of intermolecular interactions, and mobile moieties that promote disorder during self‐assembly. These structural features work synergistically to enhance the molecule ability to form robust and functional gels.

## Supporting Information

The authors have cited additional references within the Supporting Information.[^72^, ^73^, ^74^, ^75^, ^76^, ^77^, ^78^, ^79^, ^80^, ^81^] Supplementary Information available: Synthetic procedures and characterization for the preparation of Lau‐Phe‐OH, Pal‐Phe‐OH and Az‐(Phe‐OH)_2_; Methodology for the determination of the apparent pK_a_; XRD analyses; Experimental and calculated powder XRD pattern and difference profile of Pal‐L‐Phe‐OH; Crystal data and refinement details for crystalline Lau‐Phe‐OH, Pal‐Phe‐OH and Az‐(Phe‐OH)_2_; Experimental and calculated powder XRD patterns for compounds Lau‐Phe‐OH and Az‐(Phe‐OH)_2_; Thermogram recorded on a polycrystalline sample of compound Pal‐Phe‐OH; Procedure for Gel Preparation; Procedure for the Study of the Minimum Gelation Concentration (MGC); Photographs of the trials for the measurement of the MGC; Amplitude sweep test of the hydrogels of Pal‐Phe‐OH at different concentrations; ECD and UV spectra recorded on 0.2 % w/V MeOH solutions of Lau‐Phe‐OH and Pal‐Phe‐OH; ECD spectra recorded on 0.5 % w/V solutions of Lau‐Phe‐OH and Pal‐Phe‐OH in methanol and alkaline water; ECD spectra recorded on 0.5 % w/V samples of Lau‐Phe‐OH and Pal‐Phe‐OH at different times.

## Conflict of Interests

The authors declare no conflict of interest.

1

## Supporting information

As a service to our authors and readers, this journal provides supporting information supplied by the authors. Such materials are peer reviewed and may be re‐organized for online delivery, but are not copy‐edited or typeset. Technical support issues arising from supporting information (other than missing files) should be addressed to the authors.

Supporting Information

## Data Availability

The data that support the findings of this study are available in the supplementary material of this article.
