# Supplementary material for: Phenylalanine‐Based Amphiphilic Self‐Assembled Materials: Gels or Crystals?
Source: Chemistry. 2025 Mar 17;31(23):e202404586. doi: 10.1002/chem.202404586 (PMC12015405; doi:10.1002/chem.202404586)
Supplement: Supplementary file 1 — Supporting Information [file CHEM-31-e202404586-s001.pdf]

Chemistry–A European Journal

Supporting Information

Phenylalanine-Based Amphiphilic Self-Assembled Materials: Gels or Crystals?

Fabia Cenciarelli, Demetra Giuri, Silvia Pieraccini, Stefano Masiero, Simone D'Agostino,* and Claudia Tomasini*

SUPPORTING INFORMATION

Phenylalanine-Based Amphiphilic Self-Assembled Materials: Gels or Crystals?

Fabia Cenciarelli,[a] Demetra Giuri,[a] Silvia Pieraccini,[a] Stefano Masiero,[a] Simone D'Agostino,*[a] Claudia Tomasini*[a]

[a] *Dipartimento di Chimica Giacomo Ciamician, Università di Bologna, Via Piero Gobetti, 85, 40129 Bologna, Italy*

**Scheme S1.** Scheme of the Synthesis of Lau-Phe-OH **A** and Pal-Phe-OH **B**	Page S2
**Scheme S2.** Scheme of the Synthesis of Az-(Phe-OH)$_2$ **C**	Page S2
**Synthetic Procedures for the Preparation of Lau-Phe-OH A, Pal-Phe-OH B and Az-(Phe-OH)$_2$ C**	Pages S3-S5
IR-ATR, ^{1}H NMR, ^{13}C NMR, COSY spectra and HPLC-MS analysis of Lau-Phe-OH **A**	Pages S6-S8
IR-ATR, ^{1}H NMR, ^{13}C NMR, COSY spectra and HPLC-MS analysis of Pal-Phe-OH **B**	Pages S9-S11
IR-ATR, ^{1}H NMR, ^{13}C NMR, COSY spectra and HPLC-MS analysis of Az-(Phe-OH)$_2$ **C**	Pages S12-S14
**Methodology for the determination of the apparent pK_a**	Page S15
**Table S1**. Data for pK_a determination of Lau-Phe-OH **A**, Pal-Phe-OH **B** and Az-(Phe-OH)$_2$ **C**	Page S16
**XRD analyses**	Page S17
**Figure S1.** Experimental and calculated powder XRD pattern and difference profile of Palm-L-Phe-OH **B**	Page S17
**Table S2**. Crystal data and refinement details for crystalline Lau-Phe-OH **A**, Pal-Phe-OH **B** and Az-(Phe-OH)$_2$ **C**	Page S18
**Figure S2.** Experimental and calculated powder XRD patterns for compounds Lau-Phe-OH **A** and Az-(Phe-OH)$_2$ **C**	Page S19
**Figure S3.** Thermogram recorded on a polycrystalline sample of compound Pal-Phe-OH **B**	Page S20
**Figure S4.** Pairs of interacting molecules that contribute most to the total interaction energy in crystalline **A** and **C**.	Page S20
**Table S3.** Electrostatic, polarization, dispersion, repulsion terms and total interaction energy within the pairs of molecules shown in Figure S4.	Page S21
**Procedure for Gel Preparation**	Page S22
**Procedure for the Study of the Minimum Gelation Concentration (MGC)**	Page S22
**Figure S5.** Photographs of the trials for the measurement of the MGC	Page S23
**Procedure for the Rheological Analysis**	Page S24
**Figure S6.** Amplitude sweep test of the hydrogels of Pal-Phe-OH **B** at different concentrations	Page S24
**Procedure for the ECD analysis**	Page S25
**Figure S7.** ECD and UV spectra recorded on 0.2 % w/V MeOH solutions of Lau-Phe-OH **A** and Pal-Phe-OH **B**	Page S25
**Figure S8.** ECD spectra recorded on 0.5 % w/V solutions of Lau-Phe-OH **A** and Pal-Phe-OH **B** in methanol and alkaline water	Page S26
**Figure S9.** ECD spectra recorded on 0.5% w/V samples of Lau-Phe-OH **A** and Pal-Phe-OH **B** at different times	Page S27
**References**	Page S28

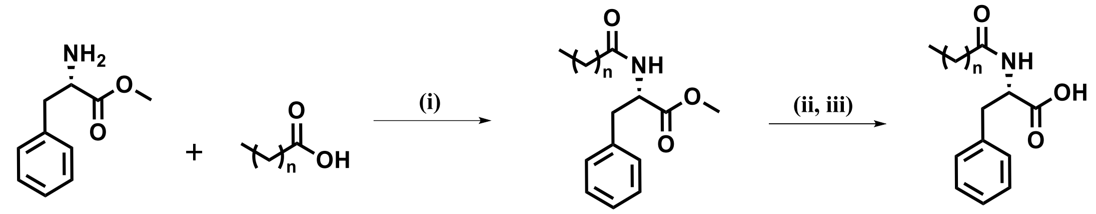

A: n=10
B: n=14

A, B

Scheme S1. Reagents and conditions: (i) fatty acid (n=10, n=14) (1 equiv.), HBTU (1.1 equiv.), DIEA (2.1 equiv.), dry ACN, r.t., 4 h; (ii) 1M NaOH (1.25 equiv.), MeOH/THF, r.t., 18 h; (iii) 1M HCl (1.35 equiv.), r.t., 10 min.

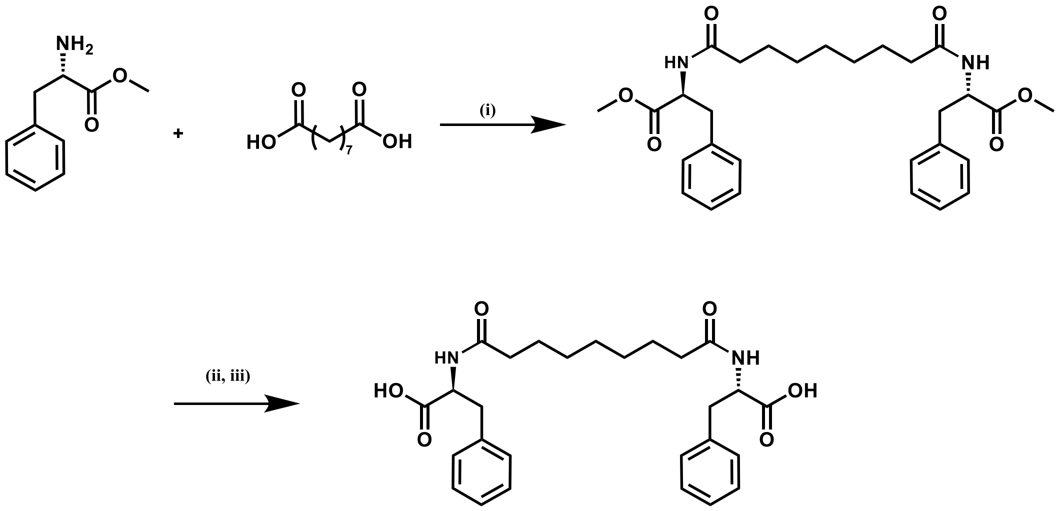

C

Scheme S2. Reagents and conditions: (i) Azelaic acid (0.5), HBTU (2.2 equiv.), DIEA (4.2 equiv.), dry ACN, r.t., 4 h; (ii) 1M NaOH (2.5 equiv.), MeOH/THF, r.t., 18 h; (iii) 1M HCl (2.7 equiv.), r.t., 10 min.

Procedures for the Preparation of A, B and C

General Remarks for the Synthetic Procedure - All reactions were carried out in dried glassware. The melting points of the compounds were determined in open capillaries and are uncorrected. All compounds were dried in vacuo and all the sample preparations were performed in a nitrogen atmosphere. High quality infrared spectra (64 scans) were obtained at 2 cm^{-1} resolution with an ATR-IR Agilent (Santa Clara, CA, USA) Cary 630 FTIR spectrometer. NMR spectra were recorded with a Varian (Palo Alto, CA, USA) Inova 400 spectrometer at 400 MHz (^{1}H NMR) and at 100 MHz (^{13}C NMR). Chemical shifts are reported in δ values relative to the solvent peak. An Agilent (Santa Clara, CA, USA) 1260 Infinity II liquid chromatograph coupled to a Mass Spectrometer MSD/XT equipped with an electrospray ionization source and operating with a single quadrupole mass analyzer was used to check the purity of compounds. The HPLC was equipped with a Phenomenex Gemini C18 −3μ—110 Å column (40 °C) and H_2O/CH_3CN with 0.2% formic acid was used as solvent. The MS was used in positive ion mode, m/z = 50–2000, fragmentor 70 V. Milli-Q water (Millipore, resistivity = 18.2 mΩ cm) was used throughout. A Jasco (Mary's Court, MD, USA) P-2000 Polarimeter was used to check the optical rotatory power of the compounds. L-Phenylalanine methyl ester hydrochloride (H-L-Phe-OMe · HCl) was purchased from TCI (>98.0%). All the solvents were purchased from Sigma-Aldrich (St. Louis, MO, USA).

Synthesis of Lau-L-Phe-OMe: In a three-neck round bottom flask the lauric acid (Lau) (200 mg, 1 mmol) was dissolved in dry ACN (9 mL) and then *O*-(Benzotriazol-1-yl)-*N,N,N',N'*-tetramethyluronium hexafluorophosphate (HBTU) (416.9 mg, 1.1 mmol) was added. The mixture was stirred at room temperature for 10 minutes. A solution containing the H-L-Phe-OMe·HCl (215.7 mg, 1 mmol) and N,N-Diisopropylethylamine (DIEA) (0.560 mL, 3.3 mmol) in dry ACN (8 mL) was then added dropwise to the first one. The mixture was stirred at r.t. and under nitrogen atmosphere for 4h, then the solvent was removed under reduced pressure and replaced with CH_2Cl_2. The organic mixture was washed with H_2O, 1M aqueous HCl, aqueous $NaHCO_3$ sat., and brine, then it was dried over Na_2SO_4 and the solvent evaporated under vacuum. The residue was purified by silica gel column chromatography to give a white solid (296.4 mg, 82.0% yield). The column eluant was a mixture of 6:1 cyclohexane:ethylacetate. Mp 56-57°C; $[\alpha]_D$ +58.8° (c = 0.5 in DCM); IR-ATR: 3311, 2918, 2849, 1757, 1733, 1641, 1543, 1472 cm^{-1}; ^{1}H-NMR (400 MHz, $CDCl_3$) δ 0.86 (3H, t, J = 8.0 Hz, Lau C*H*$_3$), 1.23-1.28 (16H, m, Lau (C*H*$_2$)$_8$), 1.52-1.59 (2H, m, $COCH_2CH_2$), 2.12-2.16 (2H, m, $COCH_2CH_2$), 3.10 (2H, ABX, J = 4.0, 8.0 Hz, CH_2Ph), 3.71 (3H, s, $COOCH_3$), 4.89 (1H, q, J = 8.0 Hz, COOC*H*), 5.88 (1H, d, J = 8.0 Hz, N*H*), 7.05-7.08 (2H, m, Phe $H_{aromatic}$), 7.20-7.29 (3H, m, Phe $H_{aromatic}$) ; ^{13}C-NMR (100 MHz, $CDCl_3$) δ 172.65, 172.18, 135.87, 129.23, 128.52, 127.08, 52.86, 52.28, 37.89, 36.54, 31.88, 29.59, 29.44, 29.31, 29.18, 25.53, 22.66, 14.10; HPLC-MS(ESI): 12.4 min; $[(M+K^++H^+)/2]$: 200.7 m/z.

Synthesis of Pal-L-Phe-OMe: In a three-neck round bottom flask the palmitic acid (Pal) (256.4 mg, 1 mmol) was dissolved in dry ACN (9 mL) and then *O*-(Benzotriazol-1-yl)-*N,N,N',N'*-tetramethyluronium

hexafluorophosphate (HBTU) (416.9 mg, 1.1 mmol) was added. The mixture was stirred at room temperature for 10 minutes. A solution containing the H-L-Phe-OMe·HCl (215.7 mg, 1 mmol) and N,N-Diisopropylethylamine (DIEA) (0.560 mL, 3.3 mmol) in dry ACN (8 mL) was then added dropwise to the first one. The mixture was stirred at r.t. and under nitrogen atmosphere for 4h, then the solvent was removed under reduced pressure and replaced with CH_2Cl_2. The organic mixture was washed with H_2O, 1M aqueous HCl, aqueous $NaHCO_3$ sat., and brine, then it was dried over Na_2SO_4 and the solvent evaporated under vacuum. The residue was purified by silica gel column chromatography to give a white solid (354.5 mg, 84.9% yield). The column eluant was a mixture of 5:1 cyclohexane:ethylacetate. Mp 73-74°C; $[\alpha]_D$ +50.4° (c = 0.5 in DCM); IR-ATR: 3855, 3747, 3736, 3678, 3650, 2922, 2851, 2359, 2344, 1748, 1645, 1541, 1466 cm^{-1}; ^{1}H-NMR (400 MHz, $CDCl_3$) δ 0.85 (3H, t, J = 8.0 Hz, Pal C*H*$_3$), 1.23-1.27 (24H, m, Pal (C*H*$_2$)$_{12}$), 1.54-1.57 (2H, m, COCH$_2$C*H$_2$*), 2.14 (2H, t, J = 8.0 Hz, COC*H$_2$*CH$_2$), 3.09 (2H, ABX, J = 4.0, 8.0 Hz, C*H$_2$*Ph), 3.70 (3H, s, COOC*H$_3$*), 4.88 (1H, q, J = 8.0 Hz, COOC*H*), 5.91 (1H, d, J = 8.0 Hz, N*H*), 7.03-7.06 (2H, m, Phe $H_{aromatic}$), 7.21-7.28 (3H, m, Phe $H_{aromatic}$) ; ^{13}C-NMR (100 MHz, $CDCl_3$) δ 172.68, 172.19, 135.88, 129.23, 128.52, 127.08, 52.87, 52.28, 37.87, 36.53, 31.91, 29.68, 29.67, 29.64, 29.61, 29.46, 29.35, 29.32, 29.19, 25.55, 22.75, 22.68, 14.12; HPLC-MS(ESI): 25.1 min; $[(M+K^++H^+)/2]$: 228.7 m/z.

Synthesis of Lau-L-Phe-OH (A): In a round bottom flask Lau-L-Phe-OMe (361.5 mg, 1 mmol) was dissolved in MeOH (1.7 mL) and THF (3.5 mL) under magnetic stirring. The whole system was placed in an ice bath in order to cool it down to 0 °C, then 1M aqueous solution of NaOH (1.25 mmol, 1.4 mL) was added. The reaction mixture was stirred at r.t. for 18h, then 1M aqueous solution of HCl (1.35 mmol, 1.5 mL) was added. The solvent was removed under reduced pressure and replaced with ethyl acetate. The organic mixture was washed with H_2O, then it was dried over Na_2SO_4 and the solvent evaporated under vacuum. A white solid was obtained (302.0 mg, 86.9% yield). Mp 98-99°C; $[\alpha]_D$ +35.1° (c = 0.5 in EtOAc); IR-ATR: 3296, 2918, 2849, 1729, 1707, 1643, 1613, 1533, 1511, 1494, 1453 cm^{-1}; ^{1}H-NMR (400 MHz, $CDCl_3$) δ 0.86 (3H, t, J = 6.4 Hz, Lau C*H$_3$*), 1.23-1.29 (16H, m, Lau (C*H*$_2$)$_8$), 1.50-1.57 (2H, m, COCH$_2$C*H$_2$*), 2.12-2.16 (2H, m, COC*H$_2$*CH$_2$), 3.17 (2H, ABX, J = 5.6, 14.0 Hz, C*H$_2$*Ph), 4.87 (1H, q, J = 7.2 Hz, COOC*H*), 6.05 (1H, d, J = 7.2 Hz, N*H*), 7.08-7.16 (2H, m, Phe $H_{aromatic}$), 7.20-7.29 (3H, m, Phe $H_{aromatic}$), 8.71 (1H, bs, COO*H*) ; ^{13}C-NMR (100 MHz, $CDCl_3$) δ 174.63, 174.03, 135.69, 129.33, 128.59, 127.17, 53.12, 37.24, 36.43, 31.89, 29.60, 29.44, 29.31, 29.28, 29.13, 25.54, 22.66, 22.39, 14.09; HPLC-MS(ESI): 10.8 min; $[M+H^+]$: 348.3, $[M+Na^+]$: 370.2 m/z.

Synthesis of Pal-L-Phe-OH (B): In a round bottom flask Pal-L-Phe-OMe (417.6 mg, 1 mmol) was dissolved in MeOH (1.7 mL) and THF (3.5 mL) under magnetic stirring. The whole system was placed in an ice bath in order to cool it down to 0°C, then 1M aqueous solution of NaOH (1.25 mmol, 1.4 mL) was added. The reaction mixture was stirred at r.t. for 18h, then 1M aqueous solution of HCl (1.35 mmol, 1.5 mL) was added. The solvent was removed under reduced pressure and replaced with ethyl acetate. The organic mixture was washed with H_2O, then it was dried over Na_2SO_4 and the solvent evaporated under vacuum. A white solid was

obtained (366.9 mg, 90.9% yield). Mp 85-86°C; $[\alpha]_D$ +31.3° (c = 0.5 in EtOAc); IR-ATR: 3280, 2912, 2849, 1707, 1654, 1546, 1470, 1425 cm^{-1}; ^{1}H-NMR (400 MHz, $CDCl_3$) δ 0.86 (3H, t, J = 6.4 Hz, Pal CH_3), 1.20-1.26 (24H, m, Pal $(CH_2)_{12}$), 1.49-1.55 (2H, m, $COCH_2CH_2$), 2.14-2.18 (2H, m, $COCH_2CH_2$), 3.17 (2H, ABX, J = 5.6, 14.0 Hz, CH_2Ph), 4.87 (1H, q, J = 7.6 Hz, COOC*H*), 6.10 (1H, d, J = 7.6 Hz, N*H*), 7.08-7.16 (3H, m, Phe $H_{aromatic}$), 7.22-7.28 (2H, m, Phe $H_{aromatic}$) 9.24 (1H, bs, COOC*H*); ^{13}C-NMR (100 MHz, $CDCl_3$) δ 174.60, 174.06, 135.70, 129.34, 128.57, 127.15, 53.11, 37.26, 36.43, 31.90, 29.68, 29.65, 29.63, 29.61, 29.46, 29.35, 29.33, 29.29, 29.14, 25.56, 22.66, 14.11, 14.08; HPLC-MS(ESI): 16.6 min; $[M-H^+]$: 402.3 m/z.

Synthesis of Az-(L-Phe-OMe)$_2$: In a three-neck round bottom flask the dicarboxylic acid (azelaic acid, Az) (94.1 mg, 0.5 mmol) was dissolved in dry ACN (5 mL) and then HBTU (416.9 mg, 1.1 mmol) was added. The mixture was stirred at room temperature for 10 minutes. A solution containing the H-L-Phe-OMe·HCl (215.7 mg, 1 mmol) and DIEA (0.560 mL, 3.3 mmol) in dry ACN (8.2 mL) was then added dropwise to the first one. The mixture was stirred at r.t. and under nitrogen atmosphere for 4h, then the solvent was removed under reduced pressure and replaced with ethyl acetate. The organic mixture was washed with H_2O (x2), 1M aqueous HCl (x3), aqueous $NaHCO_3$ sat. (x3), and brine (x2), then it was dried over Na_2SO_4 and the solvent evaporated under vacuum. The solid obtained was washed (x2) with *n*-hexane. A white solid (463.1 mg, 90.7% yield). Mp 119-121°C; $[\alpha]_D$ +75.3° (c = 0.5 in DCM); IR-ATR: 3128, 2914, 2851, 2366, 2344, 1749, 1647, 1541, 1517, 1457 cm^{-1}; ^{1}H-NMR (400 MHz, $CDCl_3$) δ 1.23-1.26 (6H, m, Az $(CH_2)_3$), 1.52-1.58 (4H, m, Az $2CH_2CH_2CO$), 2.15 (4H, t, J = 7.2 Hz, $2CH_2CH_2CO$), 3.09 (4H, ABX, J = 6.0, 14.0 Hz, $2CH_2Ph$), 3.71 (6H, s, $2CH_3COO$), 4.89 (2H, q, J = 7.6 Hz, 2C*H*COO), 6.02 (2H, d, J = 8.0 Hz, 2N*H*), 7.07-7.10 (4H, m, Phe $H_{aromatic}$), 7.21-7.30 (6H, m, Phe $H_{aromatic}$) ; ^{13}C-NMR (100 MHz, $CDCl_3$) δ 172.63, 172.22, 135.91, 129.21, 128.52, 127.07, 52.90, 52.28, 52.27, 37.87, 36.33, 28.84, 25.34; HPLC-MS(ESI): 7.5 min; $[M+H^+]$: 511.2, $[M+Na^+]$: 533.2 m/z.

Synthesis of Az-(L-Phe-OH)$_2$ (C): In a round bottom flask the Az-(L-Phe-OMe)$_2$ (510.6 mg, 1 mmol) was properly dissolved in MeOH (1.7 mL) and THF (3.5 mL) under magnetic stirring. The whole system was put in ice bath in order to cool it down to 0°C, then 1M aqueous solution of NaOH (2.5 mmol, 2.8 mL) was added. The reaction mixture was stirred at r.t. for 18h, then 1M aqueous solution of HCl (3.1 mL) was added. The solvent was removed under reduced pressure and replaced with ethyl acetate. The organic mixture was washed with H_2O, then it was dried over Na_2SO_4 and the solvent evaporated under vacuum. A white solid was obtained (426.6 mg, 88.4% yield). Mp 155-157°C;$[\alpha]_D$ +15.5° (c = 0.5 in MeOH); IR-ATR: 3300, 2912, 2851, 1707, 1641, 1526, 1420 cm^{-1}; ^{1}H-NMR (400 MHz, CD_3OD) δ 1.08-1.18 (6H, m, Az $(CH_2)_3$), 1.42-1.46 (4H, m, Az $2CH_2CH_2CO$), 2.11 (4H, t, J = 8 Hz, , $2CH_2CH_2CO$), 2.90 (2H, ABX, J = 4, 8 Hz, CH_2Ph), 3.20 (2H, ABX, J = 8, 16 Hz, CH_2Ph), 4.66 (2H, q, J = 8 Hz, 2C*H*COO), 7.15-7.26 (10H, m, Phe $H_{aromatic}$); ^{13}C-NMR (100 MHz, CD_3OD) δ 174.69, 173.47, 165.30, 137.13, 132.45, 128.81, 128.00, 126.36, 53.45, 47.39, 47.17, 46.96, 36.99, 35.30, 28.60, 28.41, 25.35; HPLC-MS(ESI): 5.2 min; $[M+H^+]$: 483.2, $[M+Na^+]$: 505.2 m/z.

IR spectrum of Lau-Phe-OH **A**

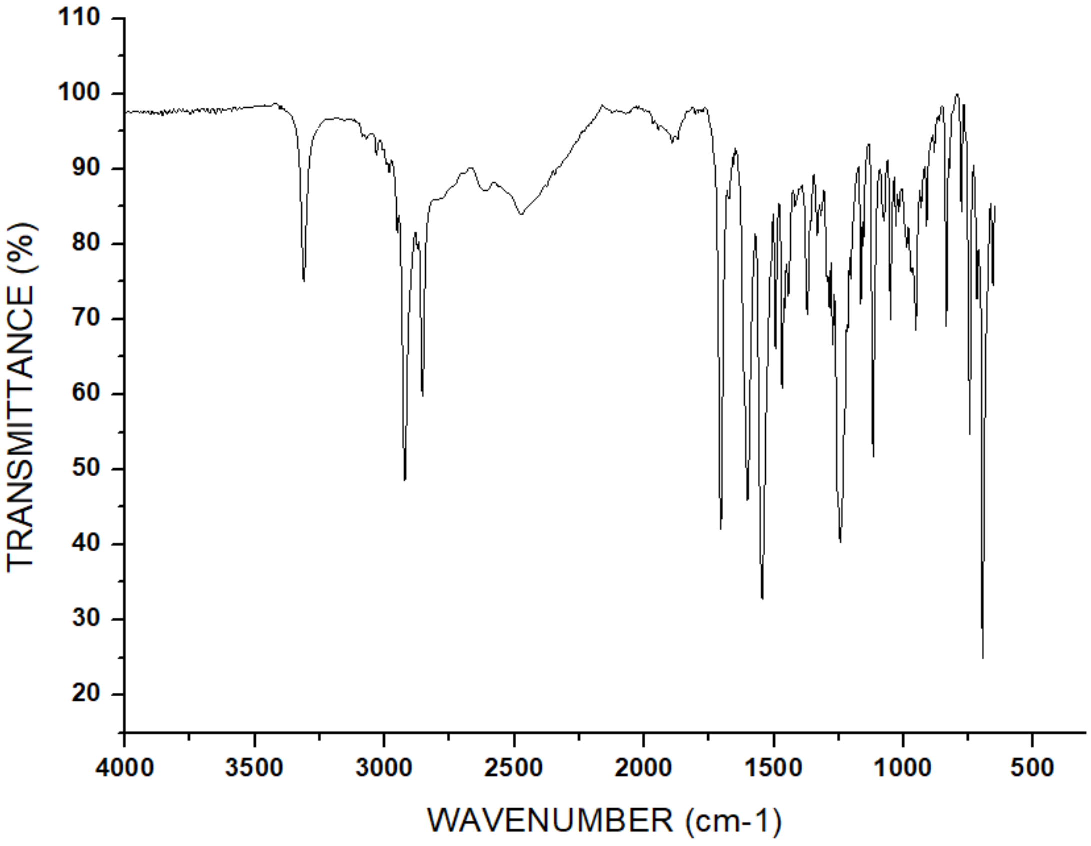

^{1}H-NMR spectrum of Lau-Phe-OH **A** ($CDCl_3$)

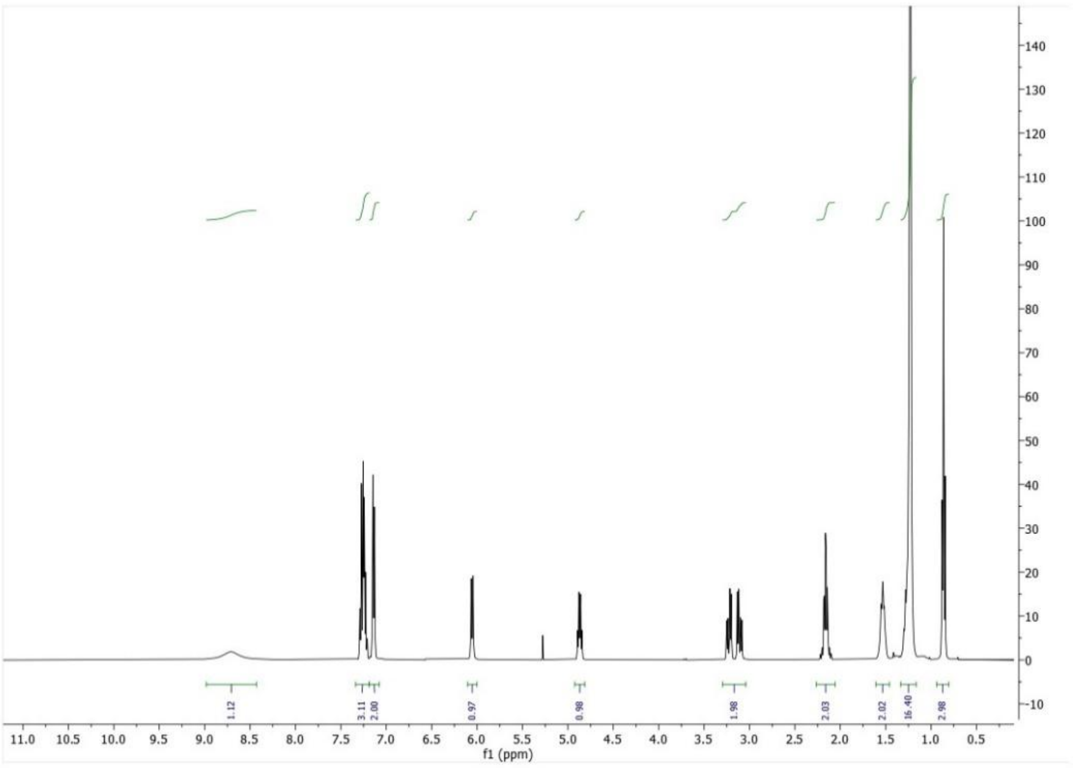

^{13}C-NMR spectrum of Lau-Phe-OH **A** ($CDCl_3$)

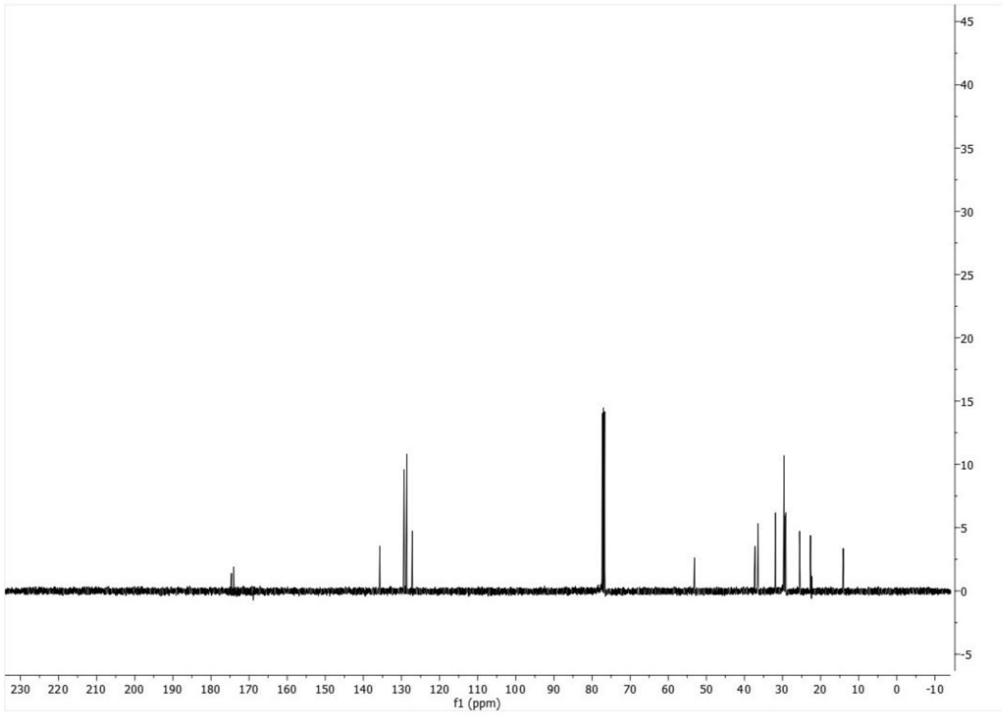

COSY spectrum of Lau-Phe-OH **A** ($CDCl_3$)

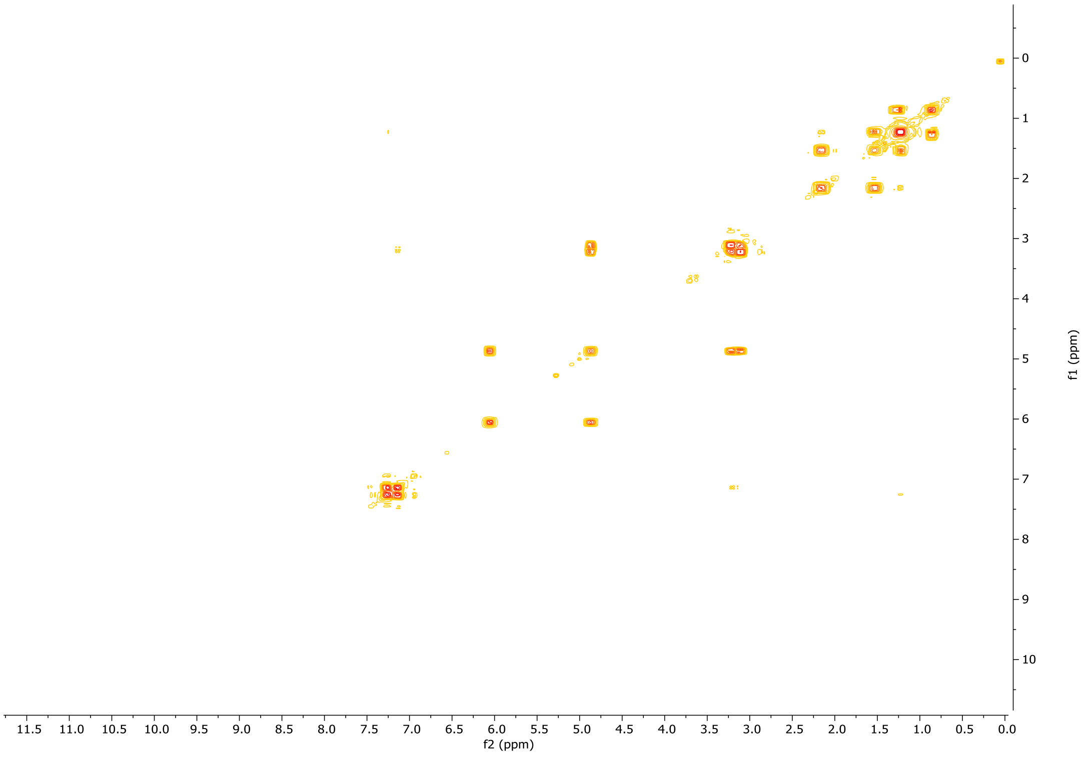

HPLC-MS of Lau-Phe-OH **A**

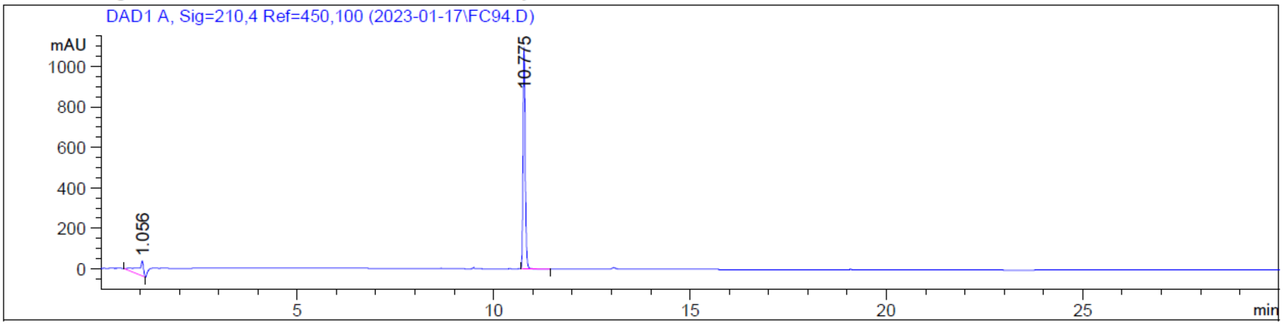

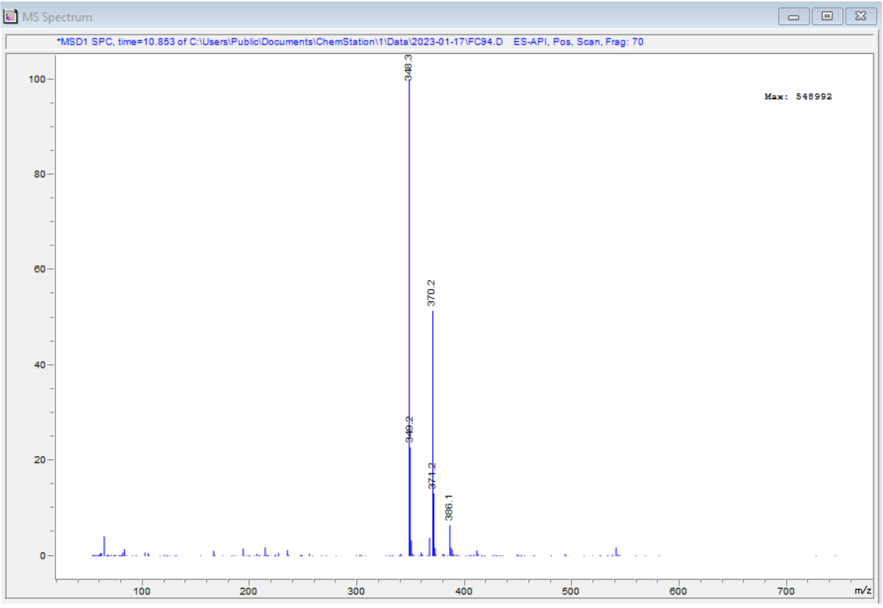

IR spectrum of Pal-Phe-OH **B**

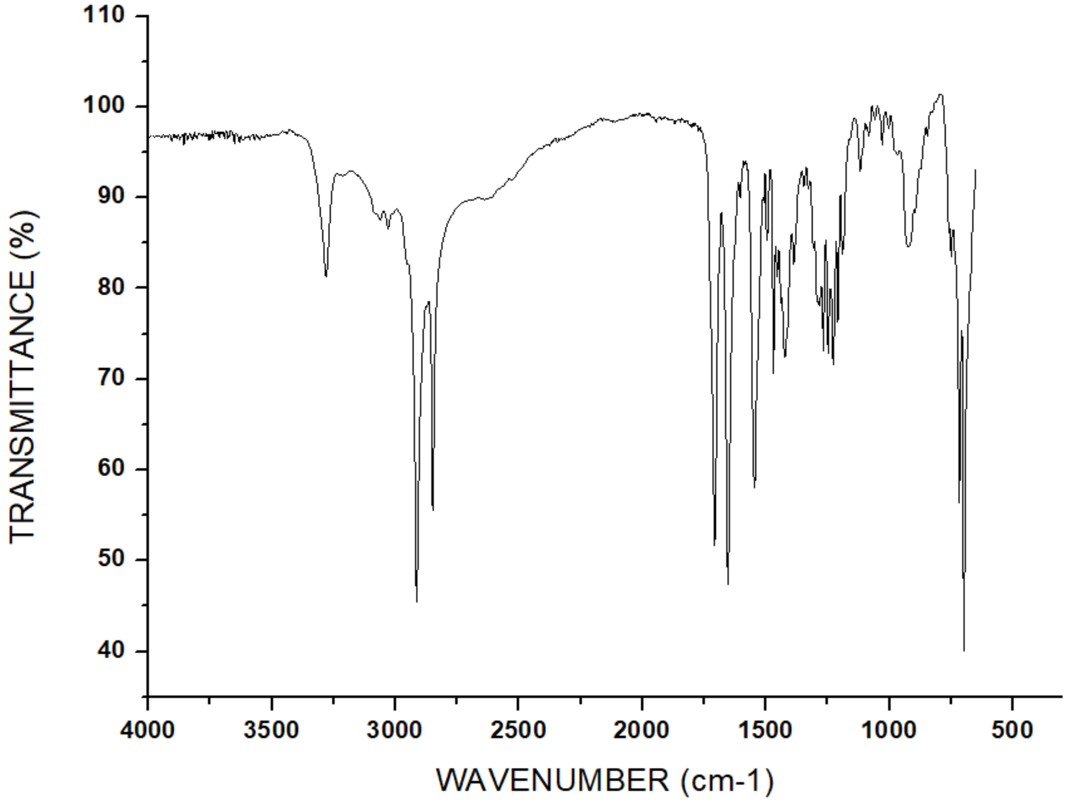

^{1}H-NMR spectrum of Pal-Phe-OH **B** ($CDCl_3$)

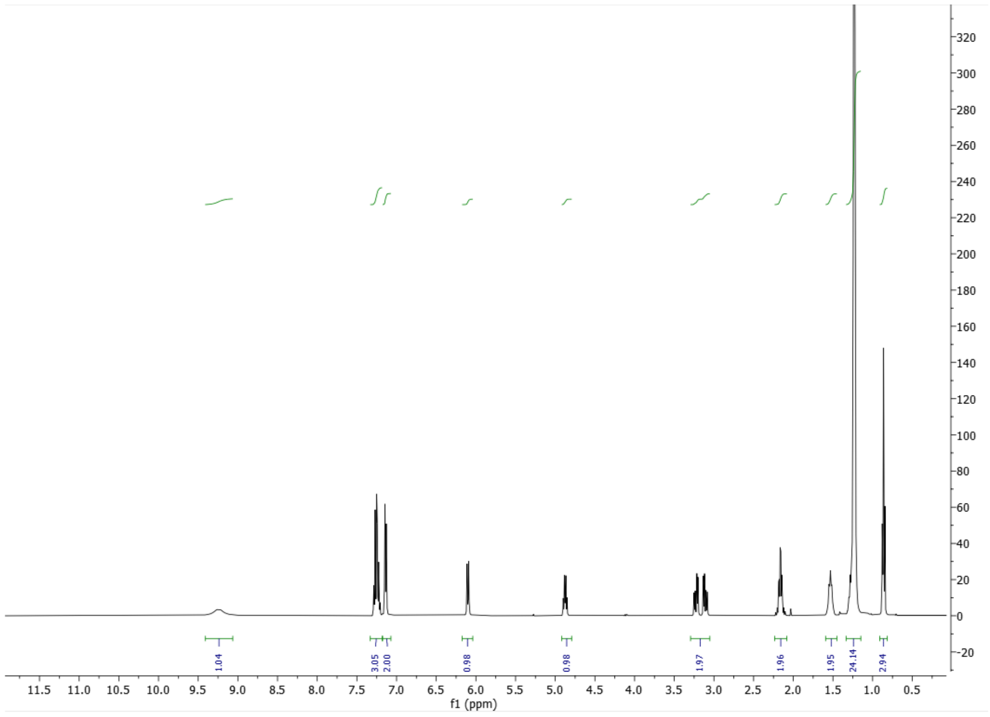

^{13}C-NMR spectrum of Pal-Phe-OH **B** ($CDCl_3$)

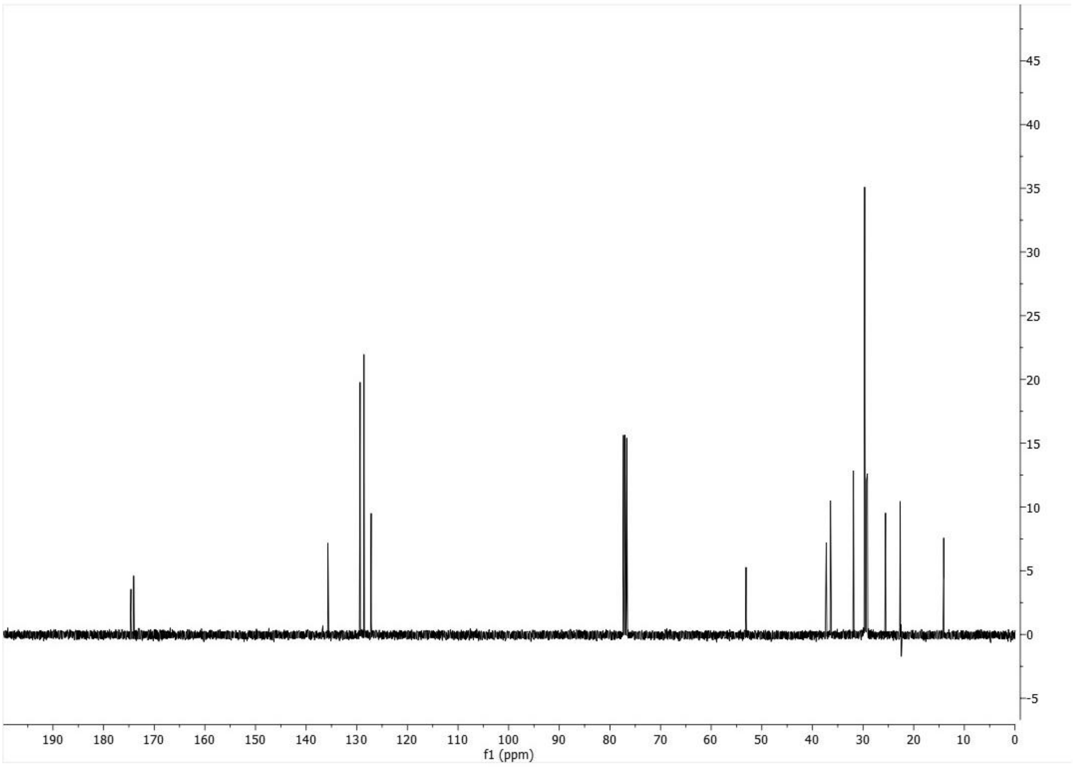

COSY spectrum of Pal-Phe-OH **B** ($CDCl_3$)

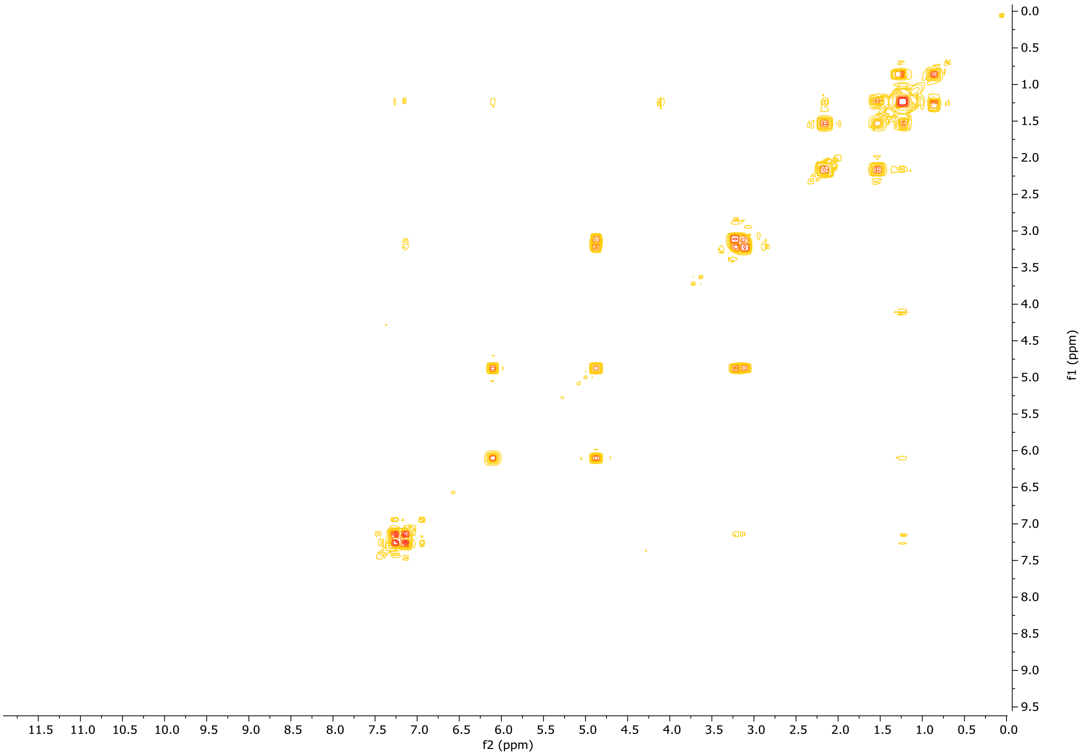

HPLC-MS of Pal-Phe-OH **B**

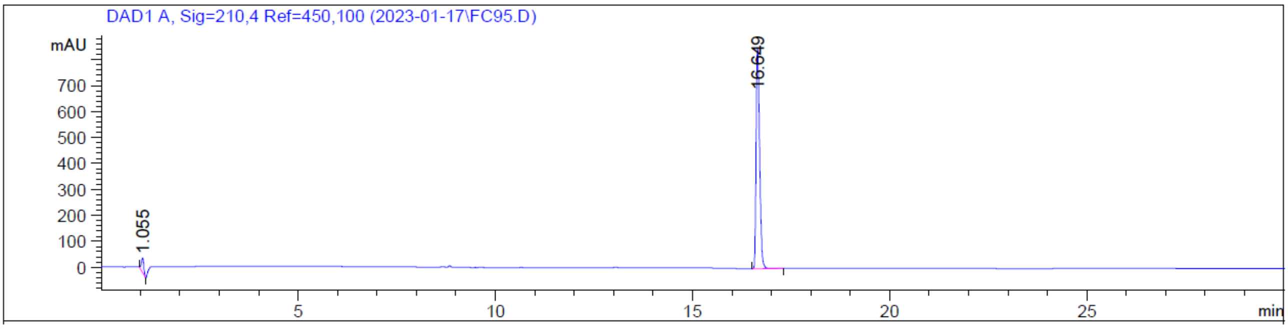

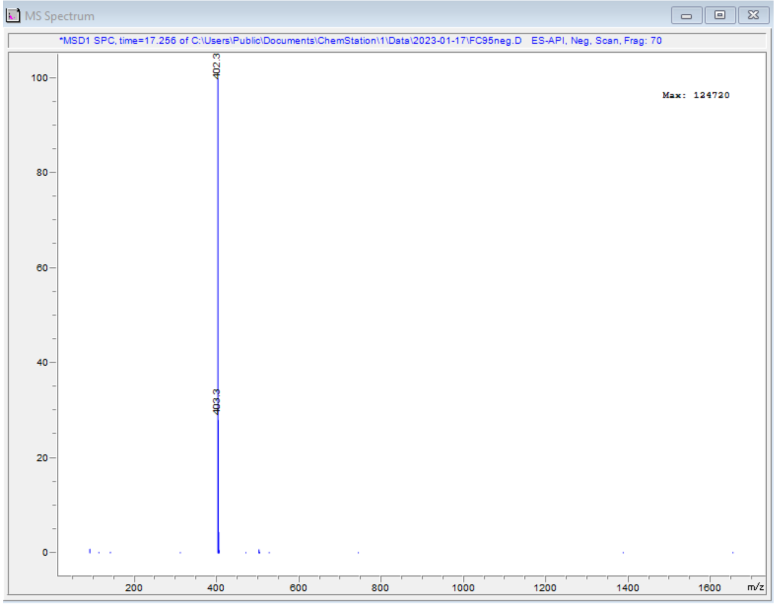

IR spectrum of Az-(Phe-OH)$_2$ **C**

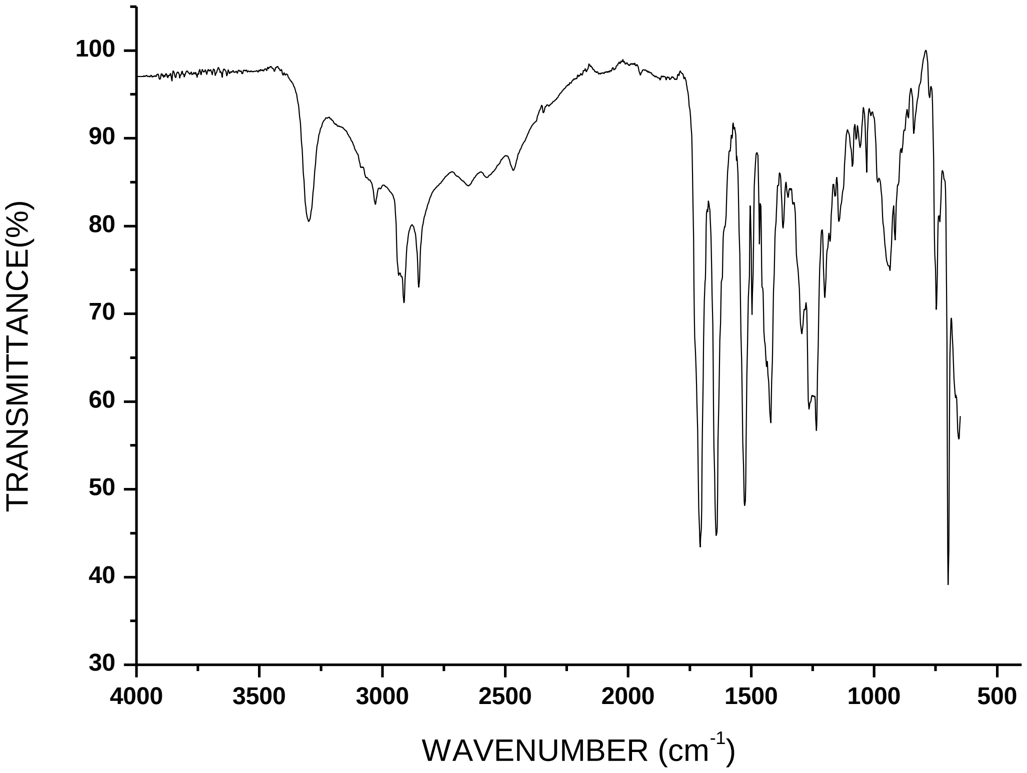

^{1}H-NMR spectrum of Az-(Phe-OH)$_2$ **C** (MeOD)

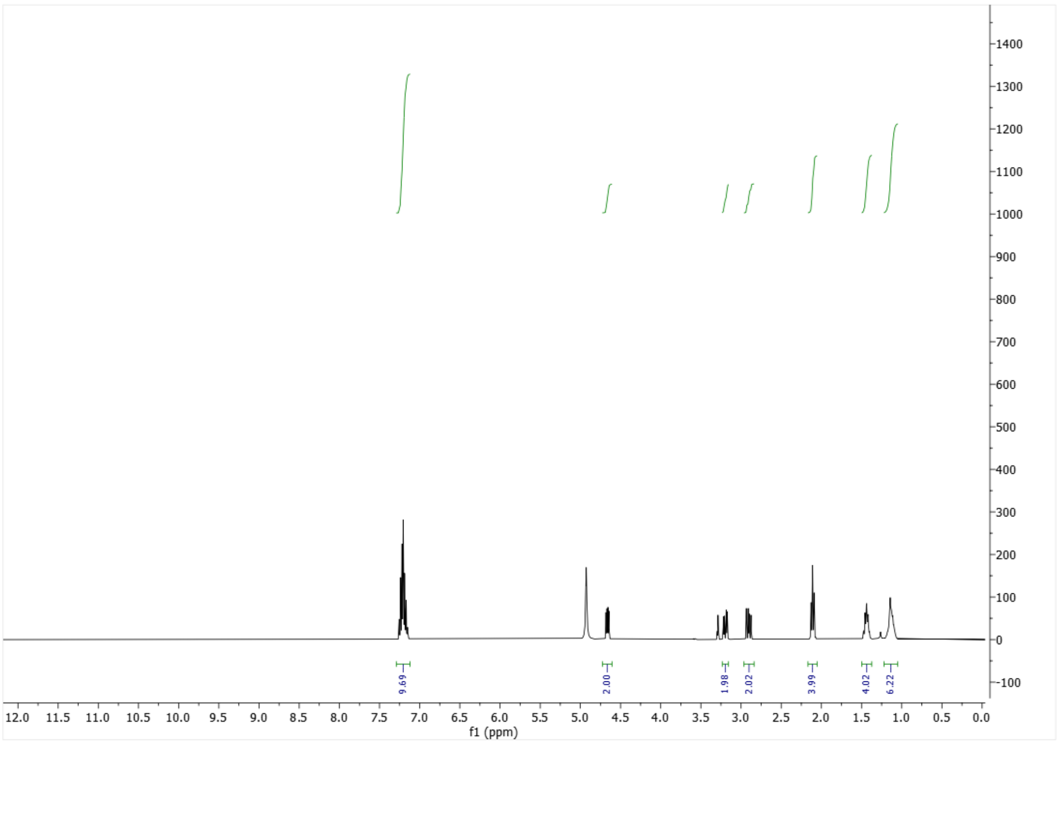

^{13}C-NMR spectrum of Az-(Phe-OH)$_2$ **C** (MeOD)

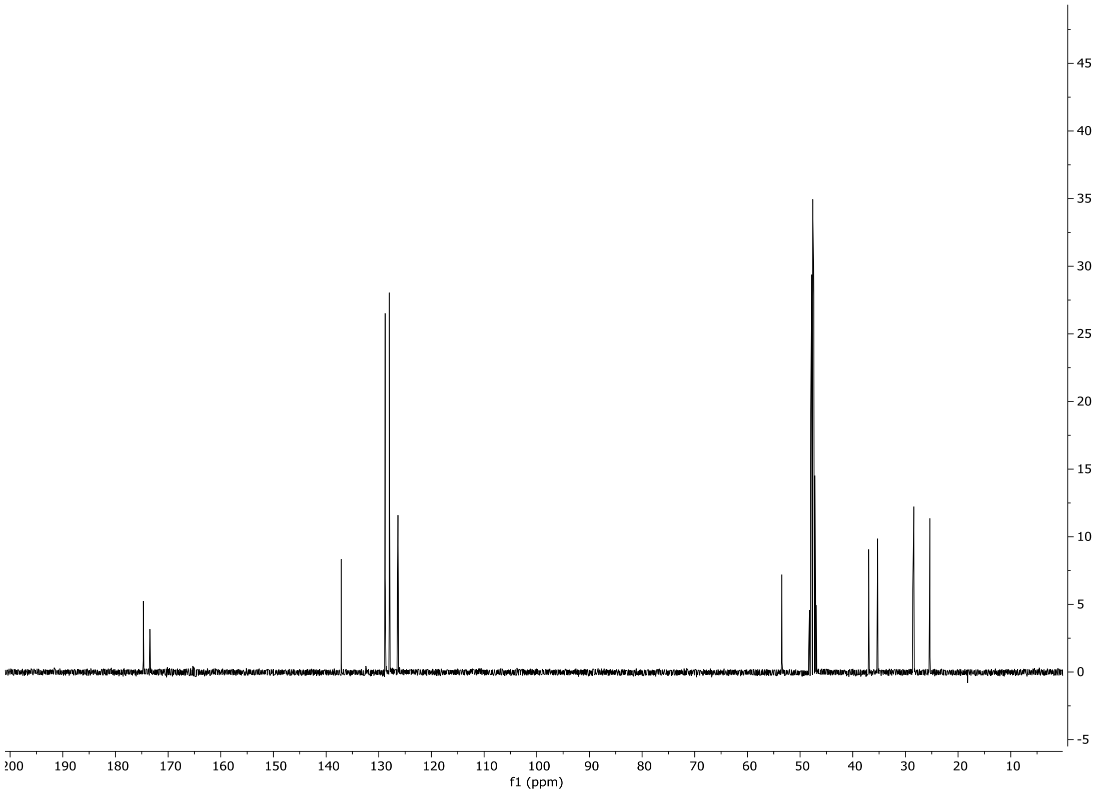

COSY spectrum of Az-(Phe-OH)$_2$ **C** (MeOD)

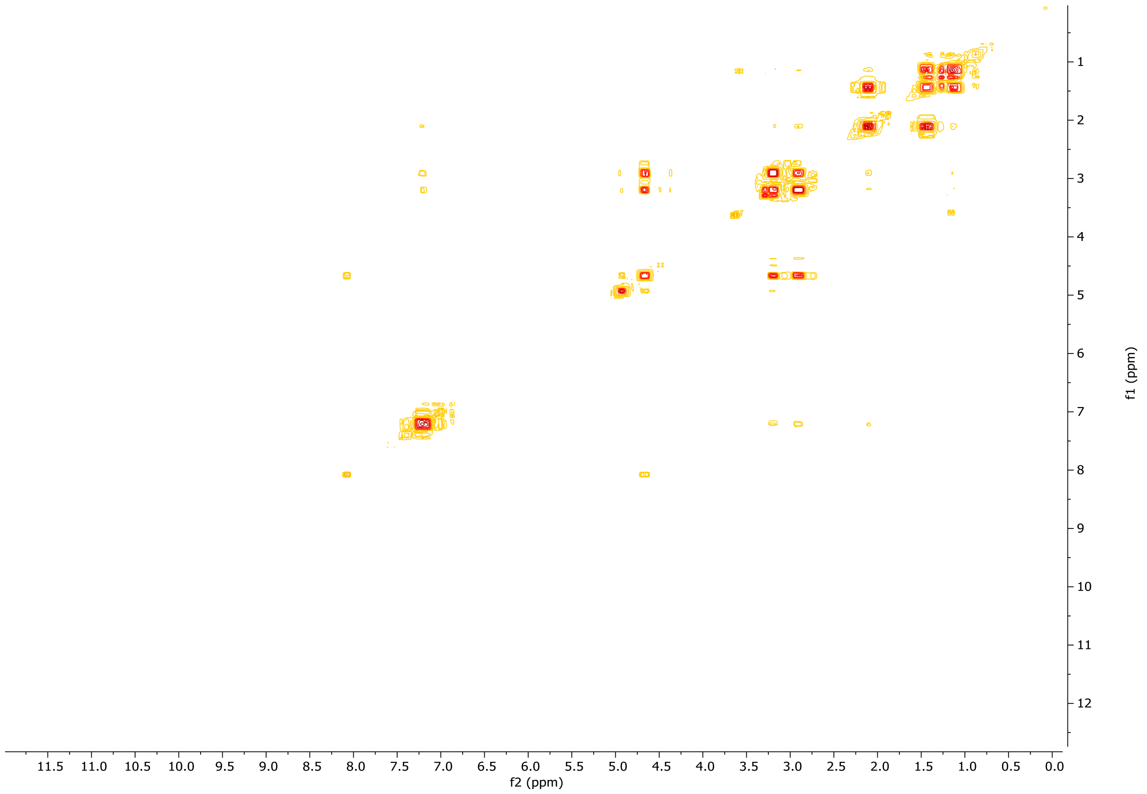

HPLC-MS of Az-(Phe-OH)$_2$ **C**

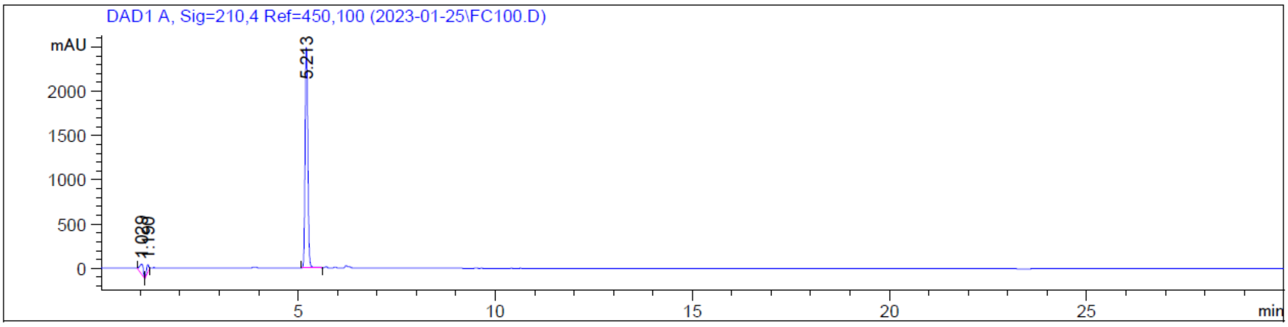

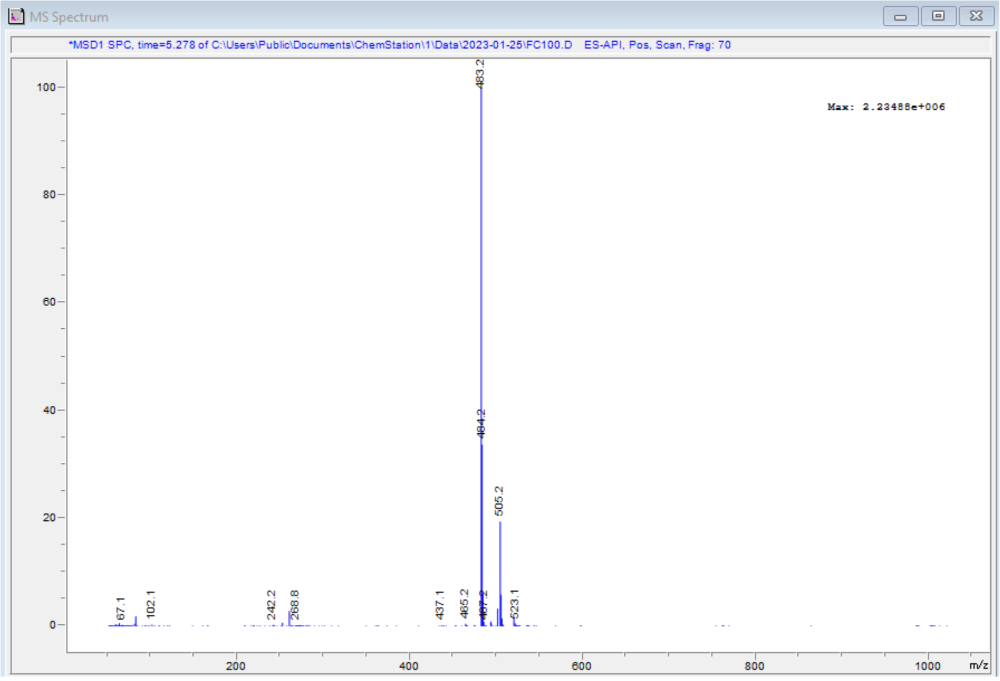

Methodology for the determination of the apparent pK_a. A XS pH70 Vio Portable pHmeter (XS Instruments, Carpi (MO), Italy) with a 2-pore steel T electrode was employed for all pH measurements. The stated accuracy of the pH measurements is ±0.1. The pH-meter was calibrated before each experiment to check the response of the electrode. Depending on the desired concentration, considering a total volume of 2 mL for each sample, the required amount of gelator was suspended into Milli-Q® H_2O. Sodium hydroxide (1 M aqueous solution) was added to the aqueous suspensions of gelator until the pH ≈ 11 was reached. The samples were vortexed and sonicated to fully dissolve the gelator. The pK_a values of gelator solutions were determined by titration via the addition of aliquots of a 0.1 M HCl solution in triplicates. pH values were recorded until reaching a stable value after each addition during the titration process. Due to the strong and constant agitation applied, samples were liquid at all times during the titration experiments.

Table S1. Data for pKa determination of Lau-Phe-OH **A**, Pal-Phe-OH **B** and Az-(Phe-OH)$_2$ **C**

LauPheOH			PalPheOH			Az(PheOH)$_2$		
mol HCl	pH		mol HCl	pH		mol HCl	pH	
0	11.11	0.01732	0	10.91	0.03464	1.50E-06	10.71667	0.05132
5.00E-07	10.84667	0.01155	5.00E-07	10.60333	0.10263	2.00E-06	10.38	0.11136
1.00E-06	10.48	0.11358	1.00E-06	10.23	0.16	2.50E-06	9.97667	0.14295
1.50E-06	10.17667	0.09292	1.50E-06	9.62333	0.39374	3.00E-06	9.26667	0.15177
2.00E-06	9.63667	0.22121	2.00E-06	8.84	0.43267	3.50E-06	7.88333	1.14583
2.50E-06	9.15667	0.08145	2.50E-06	8.14667	0.10066	4.00E-06	6.32333	0.28537
3.00E-06	7.84333	0.23861	3.00E-06	8.01667	0.03055	4.50E-06	5.56333	0.35726
3.50E-06	7.39667	0.14189	3.50E-06	8	0.06557	5.00E-06	5.27333	0.2318
4.00E-06	7.36	0.04	4.00E-06	8.08	0.08185	5.50E-06	5.10667	0.03215
4.50E-06	7.31333	0.04933	4.50E-06	8.16333	0.04509	6.00E-06	4.97667	0.08505
5.00E-06	7.33667	0.03512	5.00E-06	8.19	0.04583	7.00E-06	4.75333	0.01528
5.50E-06	7.34333	0.03512	5.50E-06	8.12	0.03	8.00E-06	4.70333	0.02517
6.00E-06	7.32333	0.05859	6.00E-06	8.08667	0.02082	9.00E-06	4.69667	0.04163
6.50E-06	7.35333	0.03215	7.00E-06	8.05667	0.06658	1.00E-05	4.76	0.05
7.00E-06	7.32333	0.03215	8.00E-06	7.87	0.02646	1.10E-05	4.67333	0.01155
7.50E-06	7.33667	0.01155	**9.00E-06**	**7.67333**	**0.06658**	1.20E-05	4.67333	0.05033
8.00E-06	7.33333	0.07371	1.00E-05	7.68333	0.01155	1.30E-05	4.67333	0.02082
8.50E-06	7.33667	0.04041	1.10E-05	7.60667	0.10066	1.40E-05	4.67	0.04359
9.00E-06	7.36	0.03606	1.20E-05	7.33333	0.05774	1.60E-05	4.63333	0.02082
1.00E-05	7.33333	0.02517	1.30E-05	7.17667	0.10066	1.80E-05	4.62333	0.03512
1.05E-05	7.34	0.03464	1.40E-05	6.68333	0.17559	**2.00E-05**	**4.64333**	**0.02082**
1.10E-05	7.31667	0.02887	1.50E-05	6.20667	0.1914	2.20E-05	4.60667	0.01528
1.15E-05	7.3	0.07	1.60E-05	5.65667	0.11372	2.40E-05	4.58	0.01732
1.20E-05	7.33	0.02646	1.70E-05	4.87667	0.11015	2.60E-05	4.55333	0.03786
1.30E-05	7.34	0.02646	1.80E-05	3.92	0.03	2.80E-05	4.52667	0.02082
**1.40E-05**	**7.3**	**0.09849**				3.00E-05	4.48667	0.04163
1.50E-05	7.16667	0.07506				3.20E-05	4.40333	0.02517
1.60E-05	7.16333	0.09074				3.40E-05	4.32667	0.03786
1.70E-05	7.22333	0.01528				3.60E-05	4.23667	0.06506
1.80E-05	6.68	0.21071				3.80E-05	3.89333	0.17156
2.00E-05	6.43	0.07				4.00E-05	3.28667	0.24132
2.20E-05	6.06667	0.08083						
2.40E-05	5.99	0.27074						
2.60E-05	5.66667	0.28676						
2.80E-05	3.33667	0.18339						

XRD analyses

Single-crystal data for compounds Lau-L-Phe-OH (**A**), and Az-(L-Phe-OH)$_2$ (**C**) were collected at RT, on an Oxford XCalibur S CCD diffractometer equipped with a graphite monochromator (Mo-Kα radiation, λ = 0.71073Å). The structures were solved with SHELXT[1] by intrinsic phasing and refined on F^2 with SHELXL[2] implemented in the Olex2 software[3] by full-matrix least squares refinement. In compound C, each carboxylic function exhibited disorder which was treated over two positions by introducing a second FVAR and with SOFs set at 0.5 of occupancy. For all compounds, H_{NH} and H_{COOH} were directly located from the electron density maps or added in calculated positions, H_{CH} atoms were added in calculated positions, all H-atoms were refined riding on their respective atoms. All non-hydrogen atoms were anisotropically refined and the rigid-body RIGU restraints applied.[4] See Table S1 for crystallographic details.

For the structural solution of Pal-L-Phe-OH (**B**), diffractograms in the 2θ range 3−70° (step size, 0.026°; time/step, 200 s; 0.02 rad soller; V × A / 40 × 40) were collected at RT on a Panalytical X'Pert PRO automated diffractometer equipped with a PIXcel detector and operated in transmission geometry (capillary spinner), using Cu–Kα radiation without monochromator in the 2θ range 3° - 70° (continuous scan mode, step size 0.0260°, counting time 889.70 s, Soller slit 0.02, antiscatter slit ¼, divergence slit ¼, 40 mA × 40 kV). Five diffraction patterns were recorded and summed to enhance the signal to noise ratio. Powder diffraction data were analyzed with the software EXPO2014,[5] which is designed to analyze both monochromatic and non-monochromatic data. Selected peaks were chosen in the 2θ range 10–50°, and a unit cell of ca 3500Å^3 and with the most plausible space group $P2_1$ was found using the algorithm N-TREOR09[6] consistent with an asymmetric unit comprised by three Palm-L-Phe-OH molecules (molecular volume was evaluated to be approximately of ca 600 Å). Thermogravimetric analysis ruled out the presence of any solvent used during crystallization (see below) Molecular fragment (asymmetric unit) was prepared from the molecular editor in EXPO2014[5] on the basis of the formerly determined crystal structure, and its molecular geometries optimized first with the molecular mechanics module (MM) by using the Universal Force Field (UFF), and then with the semiempirical method MOPac (PV7),[7] both implemented in EXPO2014.[5]Structures were solved with the simulated annealing method without H-atoms. It is worth noting that the third Palm-L-Phe-OH could not be located within the unit cell; therefore, it was excluded and the simulated annealing procedure repeated. To the so-obtained structural solution, H-atoms were added in a calculated and the structural model optimized again with MM and MOPac-PV7 before the final Rietveld refinement. An overall thermal parameter for all the atoms was adopted. See Figure S1 for the pattern difference plot and Table S1 for crystallographic details.

The program Mercury[8] was used for molecular graphics and to calculate intermolecular interactions in each crystal structure.

For phase identification purposes, X-ray powder diffractograms in the 2θ range 5-40° (step size, 0.02°; time/step, 20 s; 0.04 rad soller; 40mA x 40kV) were collected in Bragg-Brentano geometry, using Cu Kα radiation without a monochromator, on a Panalytical X'Pert PRO automated diffractometer equipped with an X'Celerator detector. The program Mercury[8] was used to calculate the X-ray powder patterns on the basis of single crystal data collected in this work. In all cases, the identity between polycrystalline samples and single crystals was always verified by comparing experimental and calculated powder diffraction patterns (See Figure S2).

Crystal data can be obtained free of charge via www.ccdc.cam.ac.uk/conts/retrieving.html (or e-mail: deposit@ccdc.cam.ac.uk); CCDC numbers 2396886-2396888.

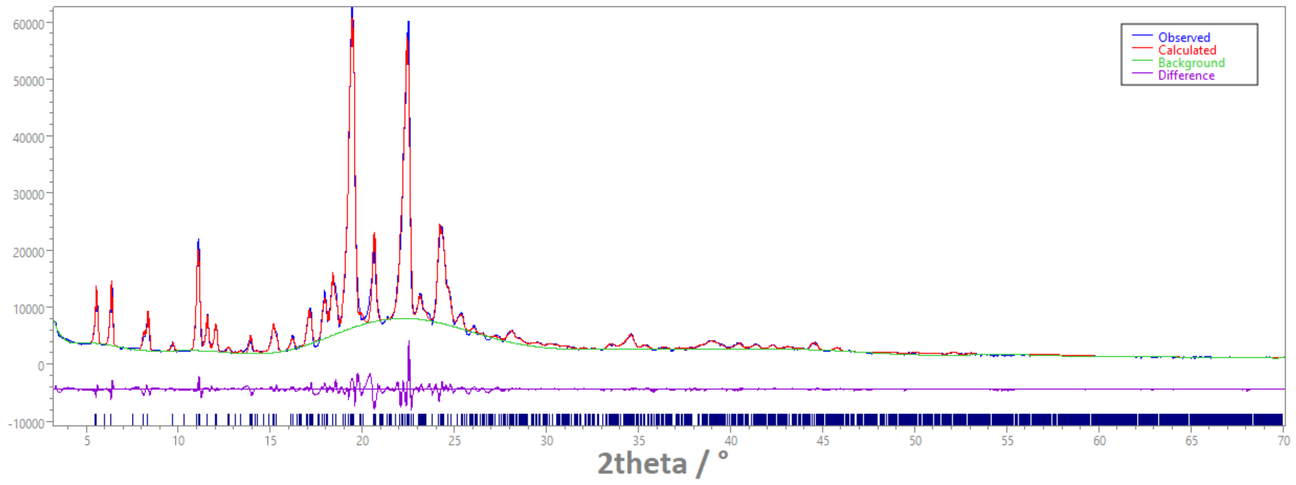

Figure S1. Experimental (blue), calculated (red) powder XRD pattern by Rietveld refinement and difference profile (magenta) of Pal-Phe-OH (**B**).

Table S2. Crystal data and refinement details for crystalline Lau-Phe-OH (**A**), Pal-Phe-OH (**B**) and Az-(Phe-OH)$_2$ (**C**).

	**A**	**B**	**C**
**Formula**	$C_{21}H_{33}NO_3$	$C_{25}H_{41}NO_3$	$C_{27}H_{33}N_2O_6$
**FW**	347.48	403.603	482.56
**Temperature (K)**	298	298	298
**Cryst. System**	Orthorhombic	Monoclinic	Monoclinic
**Space group**	$P2_12_12_1$	$P2_1$	C_2
**Z**	4	2	4
**a (Å)**	5.3683(13)	31.883(5)	17.7450(16)
**b (Å)**	13.138(2)	6.8937(13)	5.0793(6)
**c (Å)**	29.088(4)	16.073(3)	29.077(3)
**α (deg)**	90	90	90
**β (deg)**	90	93.893(4)	101.147(9)
**γ (deg)**	90	90	90
**V (Å^3)**	2051.5(7)	3524.6(11)	2571.3(5)
**D_{calc} (g/cm^3)**	1.125	0.761	1.247
**μ (mm^{-1})**	0.074	0.0381	0.088
**Measured reflections**	14814	1732	6381
**Independent reflections**	4948	1732	4462
**R_1[on F_0^2, I>2σ(I)]**	0.0865	-	0.0569
**wR_2 (all data)**	0.1718	-	0.1695
**R_{wp} (%)**	-	6.4	-

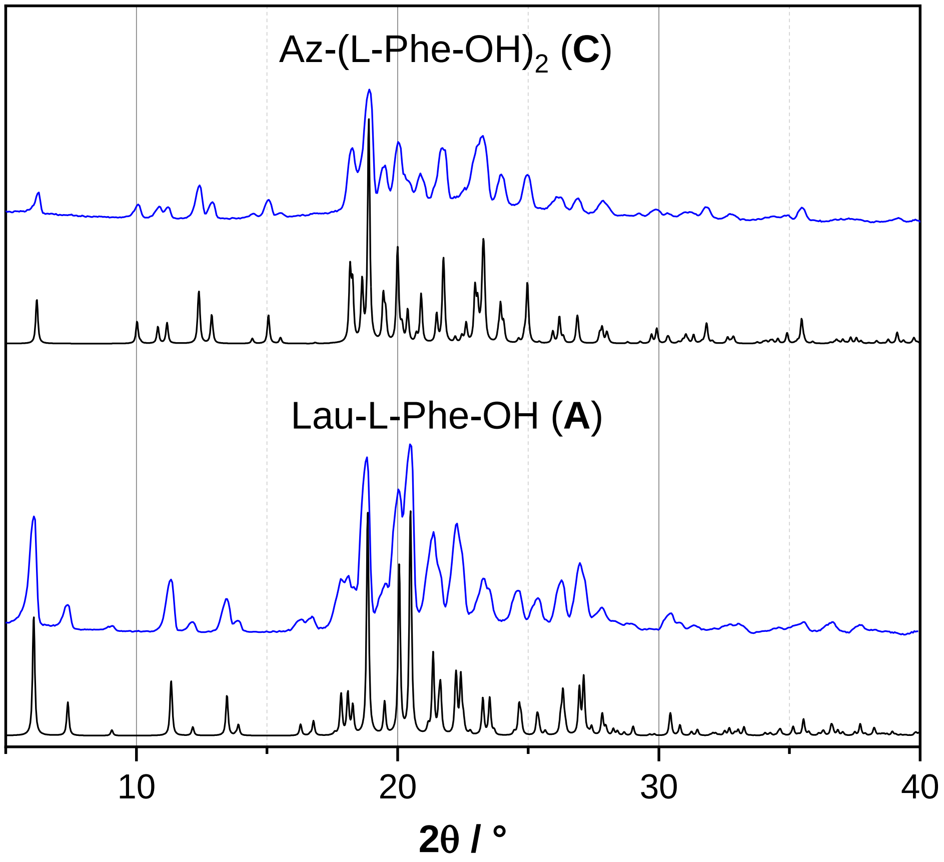

Figure S2. Experimental (black line) and calculated (black line) powder XRD patterns for compounds Lau-Phe-OH **A** and Az-(Phe-OH)$_2$ **C**.

Thermogravimetric analysis

TGA measurements on compound Pal-Phe-OH **B** was performed with a PerkinElmer TGA800 instrument in the temperature range 40–500°C under a flow of N_2 gas at a heating rate of 10.00 °Cmin^{-1}. See Figure S3.

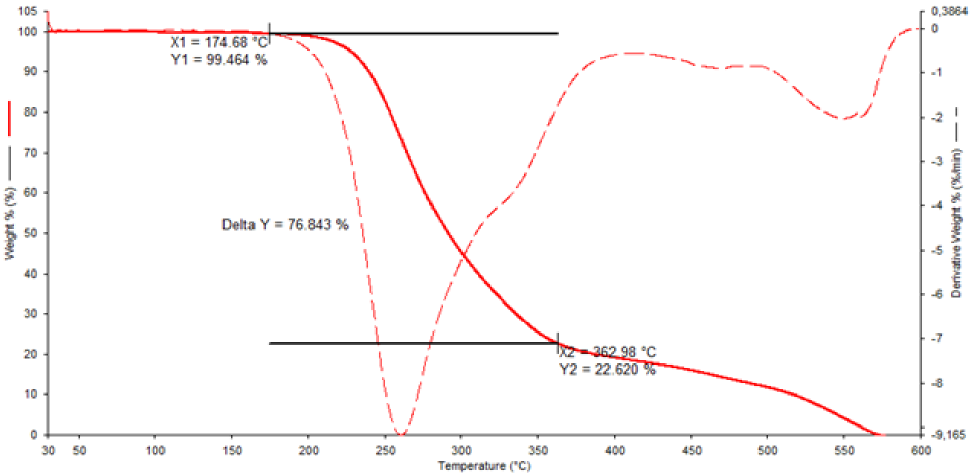

Figure S3. Thermogram recorded on a polycrystalline sample of compound Pal-Phe-OH **B**. The absence of weight loss in the region 30-200°C indicates rules out the formation of solvates/hydrates.

Hirshfeld surfaces and Intermolecular Interaction Energies (IIEs) calculation

Hirshfeld surfaces were generated for A and C based on the crystallographic information file (CIF) and Interaction energies (IIEs),[9,10] resulting from the sum of electrostatic, polarization, dispersion and repulsion contributions, were evaluated with the help of the program CrystalExplorer.[11]

Hirshfeld surface is a partition of the crystal electron density into molecular fragments, obtained essentially from the definition of a promolecule, that is a sum over the atoms of the molecule of interest of a spherically averaged electron density. On the so-obtained surface different properties can be mapped, like the distance of intermolecular interactions for example. The function d_{norm} describes the distance of any point of the surface to the closest internal (d_i) and external atoms (d_e), normalised on the Van der Waals radii of the respective atoms. It is defined as follow:

$$d_{norm} = \frac{d_i - r_i^{vdw}}{r_i^{vdw}} + \frac{d_e - r_e^{vdw}}{r_e^{vdw}}$$

A red color indicates a shorter distance than the sum of van der Waals radii relative to the closer internal and external atoms (d_{norm}<0), a blue color indicates contacts that are longer than the sum of van der Waals radii (d_{norm}>0), while white means contacts equal to the sum of van der Waals radii (d_{norm}=0).

IIEs between pairs of molecules are indicated by the same color in Table S-X and in the corresponding Figure S4.

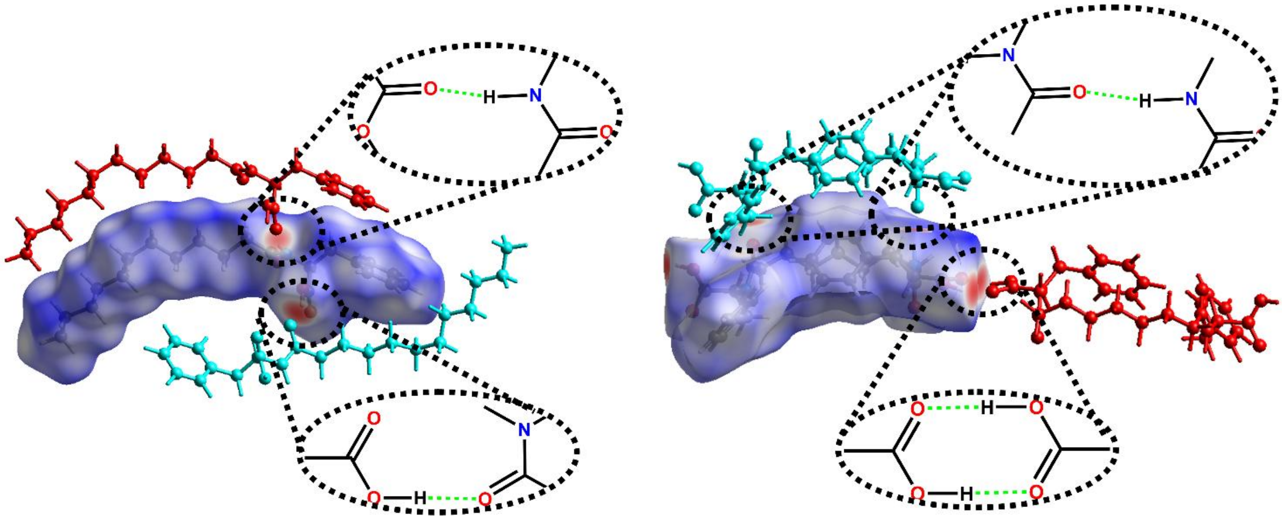

Figure S4. Pairs of interacting molecules that contribute most to the total interaction energy in crystalline A (left) and C (right). Central molecules are represented with Hirshfeld surface to highlight the main intermolecular interactions (red spots).

Table S3. Electrostatic, polarization, dispersion, repulsion terms and total interaction energy within the pairs of molecules shown in Figure S4. Evaluated with the Crystal Explorer Package and using the CE-B3LYP/6-31G(d,p) (k_ele = 1.019; k_pol = 0.651; k_disp =0.901; k_rep = 0.811) as energy model and basis set.

		R (Å)[a]	$E_{electrostatic}$ (kJ/mol)	$E_{polarization}$(kJ/mol)	$E_{dispersion}$(kJ/mol)	$E_{repulsion}$(kJ/mol)	E_{total}(kJ/mol)
**A**		5.37	-32.0	-8.9	-57.4	49.8	-56.6
		7.65	-73.9	-18.4	-29.9	88.4	-63.2
**C**		16.41	-96.8	-.34.3	-14.3	0.0	-140.1
		5.06	-75.3	-24.0	-100.4	107.4	-118.5

[a] = distance between molecular centroids (mean atomic positions).

Procedure for Gel Preparation - The gels used for the rheological analysis and the MGC studies were prepared in 7.0 mL Sterilin Cups®. The gels used for the IR spectra were directly prepared in 2 mL HPLC glass vials, using the following procedure: the gelator was suspended in Milli-Q® H_2O and 1.2 equivalents of NaOH (1M, aq) were added. The solution stirred and sonicated until complete dissolution of the gelator. To trigger the formation of the gel, solutions were added to measured quantities of glucono-δ-lactone (GdL) according to the requirements of final pH and immediately gently swirled to allow the dissolution of the trigger.

Procedure for the Study of the Minimum Gelation Concentration (MGC) - The samples were prepared by adding Milli-Q® water and aqueous 1 M NaOH (1.2 equiv) to a 7 mL Sterilin Cup containing a known quantity of the compound (**A**, **B** or **C**), in order to produce a certain final concentration of the gelator. The mixture was stirred and sonicated for about 1 h until the complete dissolution of the sample. The pH of this solution was measured to be between 10.4 and 11.3. Then glucono-δ-lactone (GdL) was added to the mixture according to the requirements of final pH that must be reached for each sample (pH ≈ 3.7 for **A** and 4.3 for **B**). After a rapid mixing and complete dissolution of GdL, the sample was allowed to stand quiescently until gel formation (overnight). After 16 h, the samples were examined by the vial inversion test.

Gelator	MGC (w/V)	Trials (w/V)
Lau-Phe-OH **A**	0.5%	0.5-0.4-0.3-0.2%
Pal-Phe-OH **B**	0.025%	0.5-0.4-0.3-0.2-0.1-0.05-0.025-0.01%
Az-(Phe-OH)$_2$ **C**	/	0.5-1.0-2.0

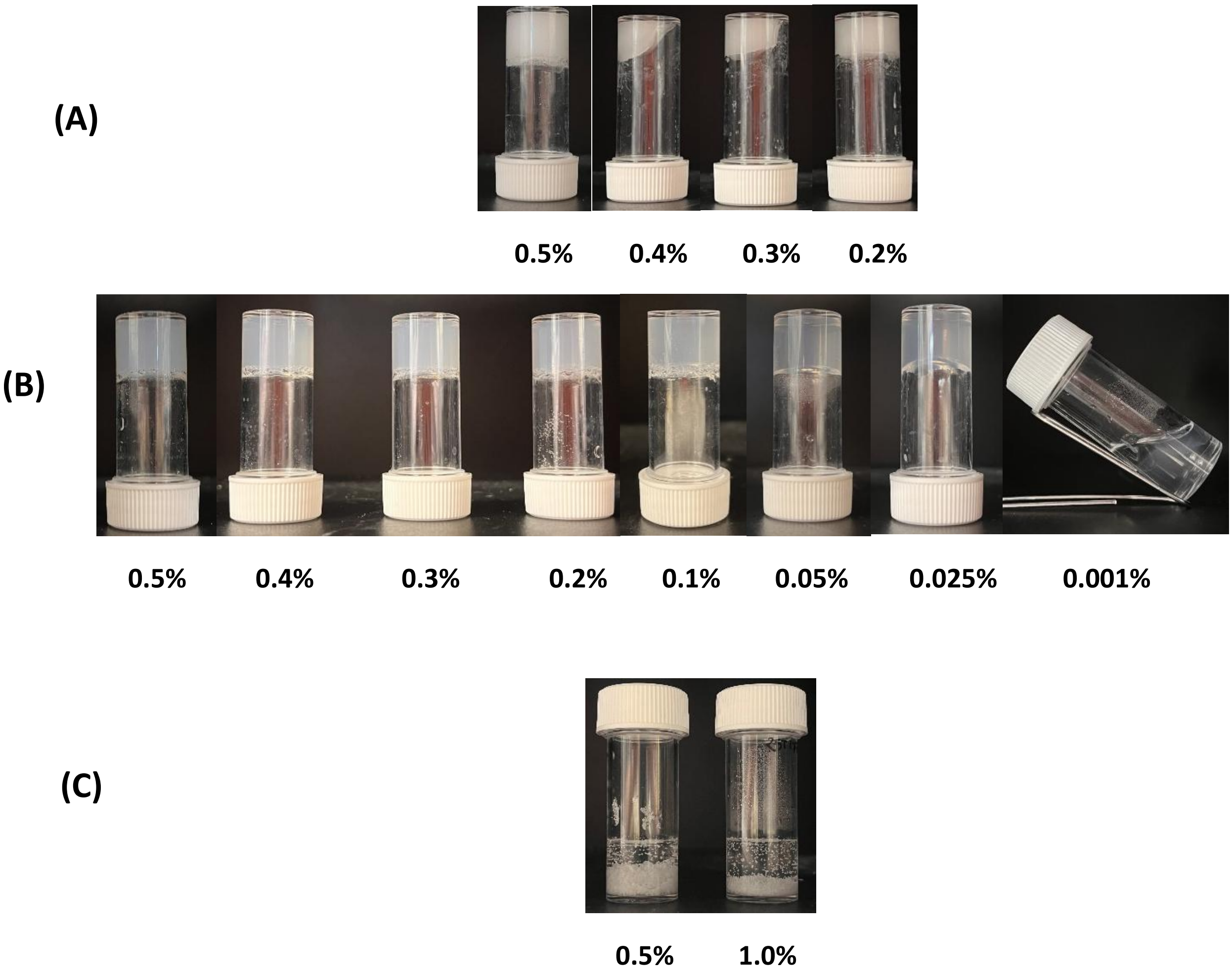

Figure S5. Photographs of the trials for the measurement of the MGC: **A)** Lau-Phe-OH, 0.5-0.4-0.3-0.2% w/V (MGC 0.5% w/V); **B)** Pal-Phe-OH, 0.5-0.4-0.3-0.2-0.1-0.05-0.025-0.01% w/V (MGC 0.025% w/V); **C)** Az-(Phe-OH)$_2$, 0.5-1.0%.

Procedure for the Rheological Analysis - The rheological measurements were performed using an Anton Paar (Graz, Austria) MCR102 rheometer. The gels (total volume of 2 mL) were directly prepared in the Thermo Fisher Scientific (Waltham, MA, USA) Sterilin cup, which fits in the rheometer. A vane and cup measuring system was used, setting a gap of 2.1 mm. Oscillatory amplitude sweep experiments (γ: 0.01–100%) were performed in triplicate, setting a temperature of 23 °C (controlled by an integrated Peltier system) using a constant angular frequency of 10 rad/s, 16 h after the addition of the trigger, to allow a complete gel formation.

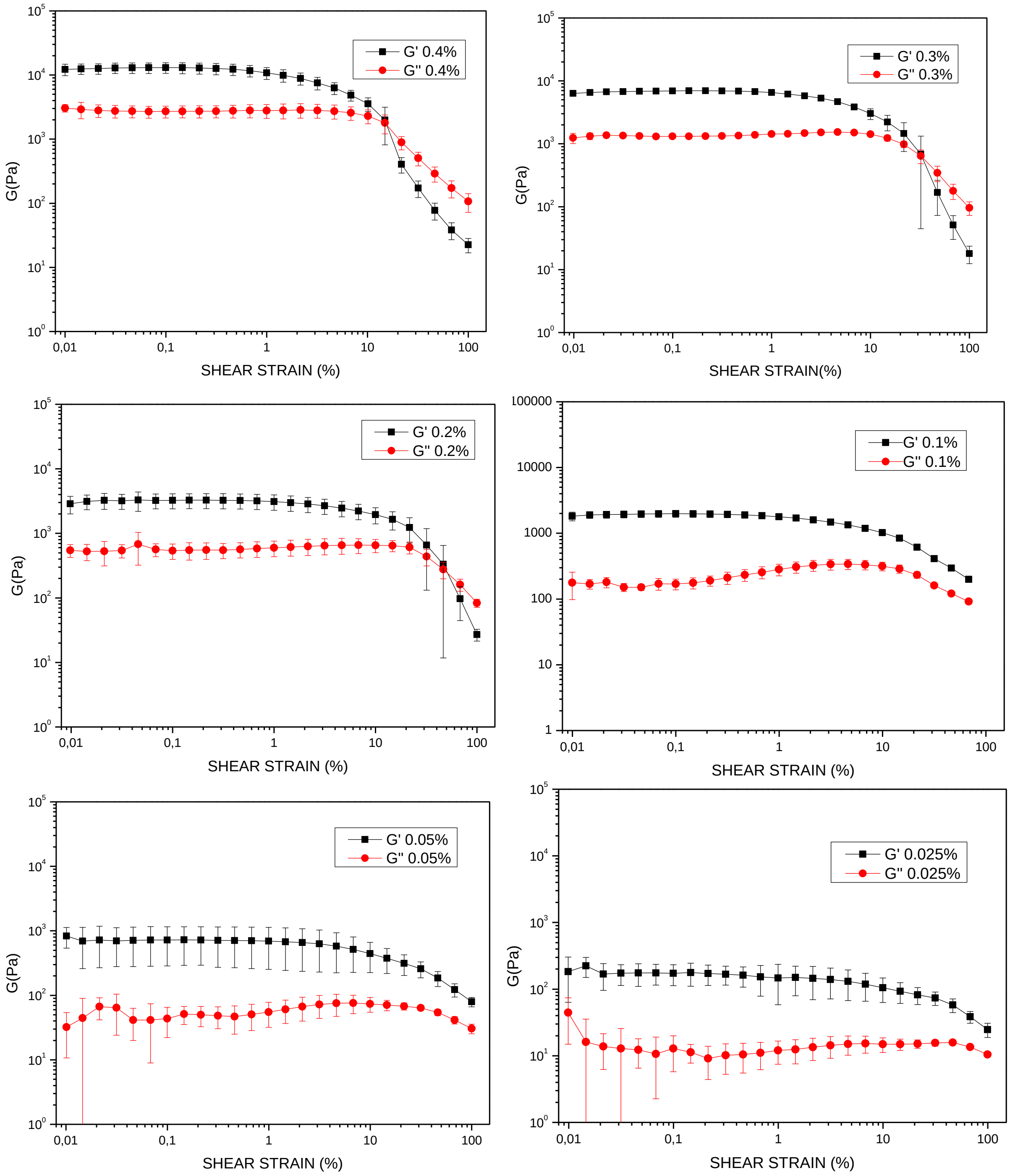

Figure S6. Amplitude sweep test of the hydrogels of Pal-Phe-OH **B** at different concentrations. The experiments were repeated in triplicate and results are expressed as mean ± standard deviation.

Procedure for the ECD Analysis. CD/UV spectra were recorded on a Jasco J-715 spectropolarimeter. Acquisitions were carried out at 100 nm/min from 400 to 185 nm, and the average of three scans was taken. For measurements on solutions 0.01 or 1 cm quartz cells were used, as specified in caption at figures. Spectra were recorded at room temperature when not otherwise specified. A Neslab RTE-111 circular thermostat (temperature stability ± 0.5°C) and cells equipped with a thermostating jacket were used for *VT*-CD measurements on solutions. Measurements on hydrogels were performed in 0.001 cm demountable sandwich cells. Each hydrogel, prepared in triplicate, was deposited and measured three times and, for each sample, the average of all collected spectra was reported. To avoid orientational effects different orientations (front, back and 90 degrees rotation) were verified.

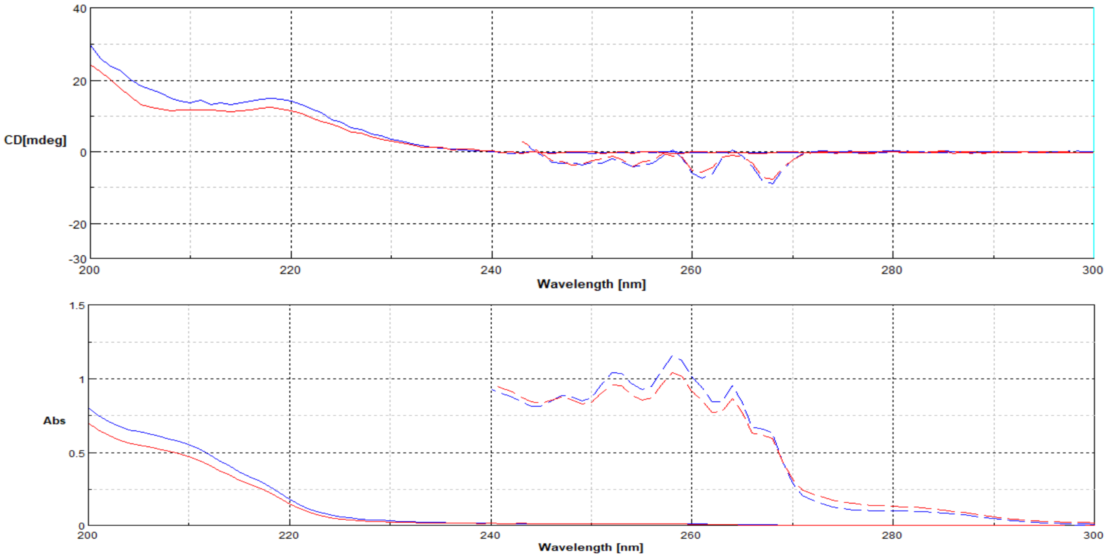

Figure S7. ECD and UV spectra recorded at room temperature on 0.2 % w/V MeOH solutions of Lau-Phe-OH **A** (5.7 mM, blue lines) and Pal-Phe-OH **B** (4.9 mM, red lines). Measurements were performed by using a 0.01 (full lines) and a 1 cm (dashed lines) path length cell. Traces recorded at 55°C (not shown) were superimposable to those obtained at room temperature for both compounds. Ellipticity is expressed in millidegrees.

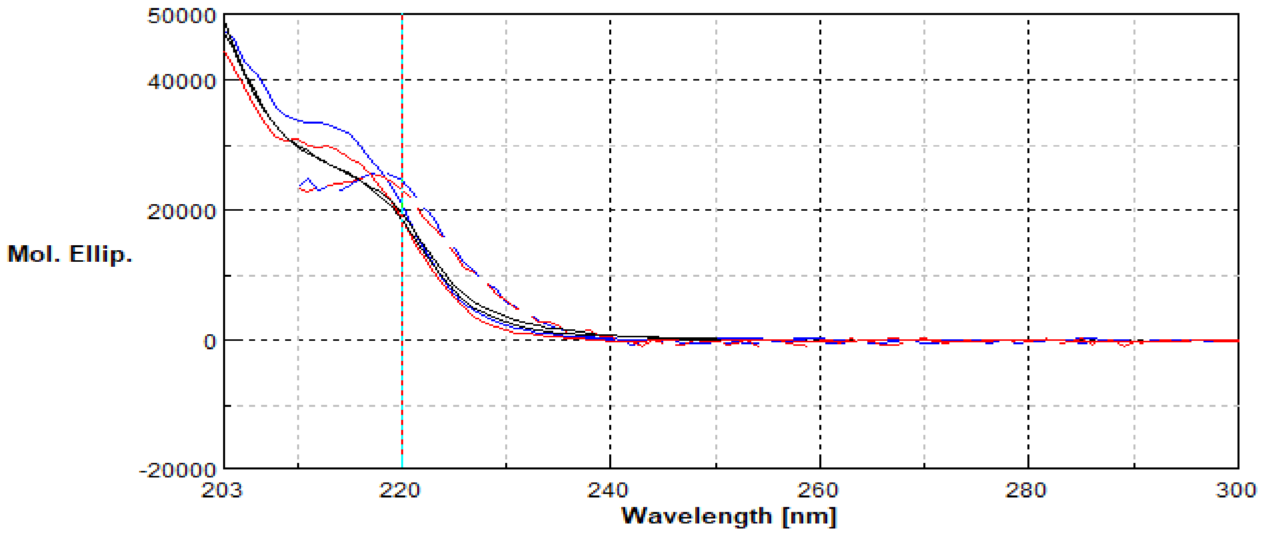

Figure S8. ECD spectra recorded on 0.5 % w/V solutions of Lau-Phe-OH **A** (14.4 mM, blue lines) and Pal-Phe-OH **B** (12.4 mM, red lines) in methanol (dashed lines) and alkaline water (full lines). The alkaline water solutions of **A** and **B** heated to 90°C yielded the superimposable traces shown in black. Measurements were performed by using a 0.01 cm path length cell. Ellipticities are expressed in molar ellipticity (deg x cm^2/dmol).

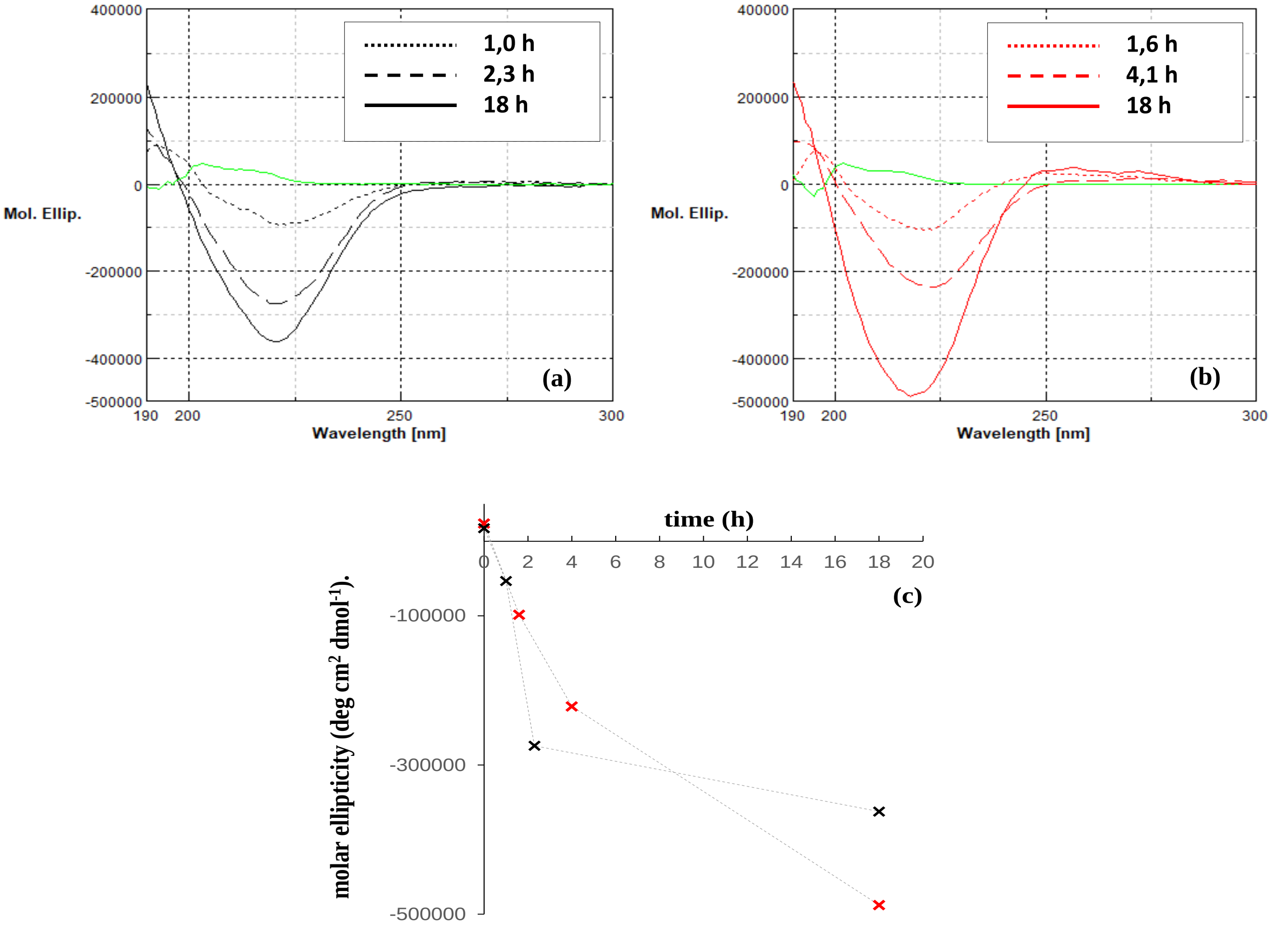

Figure S9. ECD spectra recorded on the 0.5% w/v hydrogels of Lau-Phe-OH **A** (panel **a**, in black) and Pal-Phe-OH **B** (panel **b**, in red) at different times after addition of GdL to the aqueous solution. A 0.001 cm sandwich cell was used. For the sake of comparison spectra of corresponding 0.5% w/v aqueous solutions are reported in green (pathlength = 0,01 cm). Data are expressed in molar ellipticity (deg cm^2 dmol^{-1}). In panel **(c)** molar ellipticities at 221 nm for gelator **A** (black) and 218 nm for gelator **B** (red) are plotted against times.

References

(1) Sheldrick, G. M. SHELXT - Integrated Space-Group and Crystal-Structure Determination. *Acta Crystallogr. Sect. A Found. Crystallogr.* **2015**, *71* (1), 3–8. https://doi.org/10.1107/S2053273314026370.

(2) Sheldrick, G. M. Crystal Structure Refinement with SHELXL. *Acta Crystallogr. Sect. C Struct. Chem.* **2015**, *71* (Md), 3–8. https://doi.org/10.1107/S2053229614024218.

(3) Dolomanov, O. V.; Bourhis, L. J.; Gildea, R. J.; Howard, J. A. K.; Puschmann, H. OLEX2: A Complete Structure Solution, Refinement and Analysis Program. *J. Appl. Crystallogr.* **2009**, *42* (2), 339–341. https://doi.org/10.1107/S0021889808042726.

(4) Thorn, A.; Dittrich, B.; Sheldrick, G. M. Enhanced Rigid-Bond Restraints. *Acta Crystallogr. Sect. A Found. Crystallogr.* **2012**, *68* (4), 448–451. https://doi.org/10.1107/S0108767312014535.

(5) Altomare, A.; Cuocci, C.; Giacovazzo, C.; Moliterni, A.; Rizzi, R.; Corriero, N.; Falcicchio, A. EXPO2013: A Kit of Tools for Phasing Crystal Structures from Powder Data. *J. Appl. Crystallogr.* **2013**, *46* (4), 1231–1235. https://doi.org/10.1107/S0021889813013113.

(6) Altomare, A.; Giacovazzo, C.; Guagliardi, A.; Moliterni, A. G. G.; Rizzi, R.; Werner, P. E. New Techniques for Indexing: N-TREOR in EXPO. *J. Appl. Crystallogr.* **2000**, *33* (4), 1180–1186. https://doi.org/10.1107/S0021889800006427.

(7) Fornasari, L.; Mazzaro, R.; Boanini, E.; D'Agostino, S.; Bergamini, G.; Grepioni, F.; Braga, D. Self-Assembly and Exfoliation of a Molecular Solid Based on Cooperative B-N and Hydrogen Bonds. *Cryst. Growth Des.* **2018**, *18* (12), 7259–7263. https://doi.org/10.1021/acs.cgd.8b01674.

(8) Macrae, C. F.; Sovago, I.; Cottrell, S. J.; Galek, P. T. A.; McCabe, P.; Pidcock, E.; Platings, M.; Shields, G. P.; Stevens, J. S.; Towler, M.; Wood, P. A. Mercury 4.0 : From Visualization to Analysis, Design and Prediction. *J. Appl. Crystallogr.* **2020**, *53* (1), 226–235. https://doi.org/10.1107/S1600576719014092.

(9) Spackman, M. A.; Jayatilaka, D. Hirshfeld Surface Analysis. *CrystEngComm* **2009**, *11* (1), 19–32. https://doi.org/10.1039/b818330a.

(10) Mackenzie, C. F.; Spackman, P. R.; Jayatilaka, D.; Spackman, M. A. CrystalExplorer Model Energies and Energy Frameworks: Extension to Metal Coordination Compounds, Organic Salts, Solvates and Open-Shell Systems. *IUCrJ* **2017**, *4*, 575–587. https://doi.org/10.1107/S205225251700848X.

(11) Spackman, P. R.; Turner, M. J.; McKinnon, J. J.; Wolff, S. K.; Grimwood, D. J.; Jayatilaka, D.; Spackman, M. A. CrystalExplorer: A Program for Hirshfeld Surface Analysis, Visualization and Quantitative Analysis of Molecular Crystals. *J. Appl. Crystallogr.* **2021**, *54*, 1006–1011. https://doi.org/10.1107/S1600576721002910.

(12) D. J. Adams, M. F. Butler, W. J. Frith, M. Kirkland, L. Mullen, P. Sanderson, Soft Matter 2009, 5, 1856–1862. https://doi.org/10.1039/B901556F.
